# Reducing hippocampal extracellular matrix reverses early memory deficits in a mouse model of Alzheimer’s disease

**DOI:** 10.1186/s40478-014-0076-z

**Published:** 2014-06-29

**Authors:** Marlene J Végh, Céline M Heldring, Willem Kamphuis, Sara Hijazi, Arie J Timmerman, Ka Wan Li, Pim van Nierop, Huibert D Mansvelder, Elly M Hol, August B Smit, Ronald E van Kesteren

**Affiliations:** Center for Neurogenomics and Cognitive Research, Neuroscience Campus Amsterdam, VU University, De Boelelaan 1085, 1081 HV Amsterdam, The Netherlands; Netherlands Institute for Neuroscience, Meibergdreef 47, 1105 BA Amsterdam, The Netherlands; Department of Translational Neuroscience, Brain Center Rudolf Magnus, University Medical Center Utrecht, Universiteitsweg 100, 3584 CG Utrecht, The Netherlands

**Keywords:** Alzheimer’s disease, Hippocampus, Memory, Plasticity, Extracellular matrix, Perineuronal net

## Abstract

Alzheimer’s disease is caused by increased production or reduced clearance of amyloid-β, which results in the formation amyloid-β plaques and triggers a cascade of downstream events leading to progressive neurodegeneration. The earliest clinical symptoms of Alzheimer’s disease, i.e., memory loss, are however poorly understood from a molecular and cellular perspective. Here we used APPswe/PS1dE9 (APP/PS1) transgenic mice to study the early pre-pathological effects of increased amyloid-β levels on hippocampal synaptic plasticity and memory. Using an unbiased proteomics approach we show that the early increase in amyloid-β levels in APP/PS1 mice at three months of age coincides with a robust and significant upregulation of several protein components of the extracellular matrix in hippocampal synaptosome preparations. This increase in extracellular matrix levels occurred well before the onset of plaque formation and was paralleled by impairments in hippocampal long-term potentiation and contextual memory. Direct injection into the hippocampus of the extracellular matrix inactivating enzyme chondroitinase ABC restored both long-term potentiation and contextual memory performance. These findings indicate an important role for the extracellular matrix in causing early memory loss in Alzheimer’s disease.

**Electronic supplementary material:**

The online version of this article (doi:10.1186/s40478-014-0076-z) contains supplementary material, which is available to authorized users.

## Introduction

Alzheimer’s disease (AD) is characterized by progressive cognitive decline, with memory loss being one of the earliest clinical symptoms. The neuropathological hallmark of AD is the presence of diffuse and neuritic plaques composed of amyloid-β (Aβ) peptides formed by proteolytic cleavage of the amyloid precursor protein (APP) by β- and γ-secretases [1]. Increased production or reduced clearance of Aβ is considered a prerequisite for the neuropathological and clinical manifestation of AD [2,3]. However, the initial memory impairments in AD patients are not temporally correlated with the formation of Aβ plaques in brain areas that are important for memory processing, such as the hippocampus, and the mechanisms underlying memory loss in AD remain unclear [4].

The hippocampus plays a central role in early memory loss in AD patients [5]. The earliest neuropathological changes in AD are consistently observed in the medial temporal lobe (entorhinal cortex and hippocampus) [6], and hippocampal volume loss is the best established diagnostic marker for AD and highly predictive of disease progression [7]. Early deficits in hippocampal memory performance and synaptic plasticity have been established in various animal models of AD, before neuropathological changes are observed and in the absence of neurodegeneration [8,9]. Hippocampal synaptic dysfunction thus most likely underlies initial memory deficits in AD and may trigger further disease progression [10–13].

To identify mechanisms that contribute to hippocampal synaptic dysfunction in AD we performed an unbiased synapse-oriented proteomics screen in APPswe/PS1dE9 (APP/PS1) transgenic mice [14]. APP/PS1 have increased Aβ production resulting from the introduction of two human disease-related mutations, one in *APP* and one in presenilin 1 (*PSEN1*). Although transgenic mouse models that overproduce mutant APP generally fail to reproduce the full spectrum of pathological and clinical symptoms observed in AD, they are useful for studying early pre-pathological memory and plasticity impairments due to increased β-amyloidosis [9,15–19]. Here we report that early memory and plasticity deficits in 3 months old APP/PS1 mice coincide with an increase in hippocampal extracellular matrix (ECM) levels and are reversed by local enzymatic digestion of the ECM. Our findings thus highlight the ECM as a novel potential target in the treatment of early cognitive decline in AD.

## Materials and methods

### Animals

For details about APP/PS1 mice see The Jackson Laboratory (strain B6C3-Tg(APPswe,PSEN1dE9)85Dbo/J; stock number 004462; http://jaxmice.jax.org/). APP/PS1 transgenic mice express a chimeric mouse/human APP gene harboring the Swedish double mutation K595N/M596L (APPswe) and a human PS1 gene harboring the exon 9 deletion (PS1dE9), both under the control of the mouse prion protein promoter (MoPrP.Xho) [14,20,21]. Mice were maintained as hemizygous and crossed with wildtype C57BL/6 mice. All experiments were performed with male mice and approved by the animal ethics committee of either the Royal Netherlands Academy of Arts and Sciences or the VU University Amsterdam.

### Proteomics analysis

Details can be found in the Additional file 1. In short, synaptosomes were isolated from hippocampi of APP/PS1 mice and wildtype littermates at 1.5, 3, 6 and 12 months of age as described previously [22,23]. Five 8-plex iTRAQ experiments (i.e., five biological replicates per age group per genotype) were performed. Samples were analyzed using an ABI 5800 proteomics analyzer (Applied Biosystems, Forster City, CA). Protein identification and quantification were performed as described [24]. Mascot (MatrixScience, version 2.3.01) searches were performed against Swissprot (version 20/10/2010) and NCBInr (version 20/10/2010) databases. Proteins were considered for quantification if at least three peptides were identified in three replicate iTRAQ sets and at least one peptide in all other sets. Protein abundance was determined by taking the average normalized standardized iTRAQ peak area of all unique peptides annotated to that protein. Statistical significance was determined by calculating permutation-derived false discovery rates (FDR) using the SAM [25] package in MeV (version 4.6.1) [25,26]. Changes in protein expression were considered to be significant when the FDR is <10% and log-fold change >0.125.

### Functional protein group analysis

The Functional Classification Tool in the Database for Annotation, Visualization and Integrated Discovery (DAVID; http://david.abcc.ncifcrf.gov/) [27,28] was used to group proteins based on functional similarity and to determine enrichment of functional protein groups within the total set of differentially expressed proteins. Enrichment was determined using the total set of all detected proteins as the background set, and using the following settings: Similarity Term Overlap: 4; Similarity Threshold: 0.35; Initial Group Membership: 4; Final Group Membership: 4; Multiple Linkage Threshold: 0.50. Significance was determined based on the Fisher’s exact *p*-values reported by DAVID.

### Immunoblotting

Immunoblot analysis was performed on six independent synaptosome protein extracts. To facilitate detection of ECM proteins, protein pellets were treated with chondroitinase ABC (Sigma Aldrich, Zwijndrecht, The Netherlands) at 0.5 U/50 mg protein for 90 min at 37°C. Of each sample, 10 μg protein was mixed with SDS sample buffer and heated at 90°C for 5 min. Proteins were separated on a Criterion™ TGX Stain-Free Precast Gel (4-16% Tris-Glycine; Bio-Rad) in a Criterion™ Cell Electrophoresis System (Bio-Rad), and electroblotted onto PVDF membrane overnight at 4°C. After blocking with 5% (v/v) non-fat dry milk and 0.5% (v/v) Tween-20 in Tris-buffered saline (TBS) for 1 h at RT, blots were incubated with primary antibodies, followed by a horseradish peroxidase-conjugated secondary antibody (Dako, Glostrup, Denmark; 1:10000). The following antibodies were used: 6E10 (Signet; 1:15000), anti-Brevican (gift from Dr. C. Seidenbecher, Magdeburg, Germany; 1:2000), anti-Tenascin-R (P.Glia, Bonn, Germany; 1:2000), anti-Hapln1 (Abcam; 1:1000), anti-Neurocan (Sigma; 1:1000). Blots were incubated with ECL substrate (GE Healthcare, Pollards Wood, UK), scanned with a Odyssey Imager (LI-COR) and analyzed with Image Studio software (LI-COR, version 1.1.7) using background correction. To correct for differences in sample input, all gels were imaged before electroblotting and the total protein densitometric values were used for sample normalization, which is more reliable then normalizing to the levels of a single protein [29,30]. Significance was determined using a Student’s *t*-test.

### Immunohistochemistry

Immunohistochemistry on brain sections at 3, 6, 9 and 12 months of age was performed as previously described [31]. Brains were fixed by transcardial perfusion with 4% (v/v) paraformaldehyde in phosphate buffered saline (PBS), pH 7.0. Coronal cryosections (10 μm) were thaw-mounted on Superfrost Plus slides, dried for 1 h at RT and stored at −20°C until use. Sections were fixed with fresh 4% (v/v) paraformaldehyde in 0.1 M PBS, pH 7.0, for 10 min at RT. Sections were treated with 10 mM sodium citrate and 0.05% (v/v) Tween-20 (pH 6.0) for 20 min at 95°C, blocked with 10% (v/v) normal donkey serum and 0.4% (v/v) Triton X-100 in PBS, followed by incubation overnight with 6E10 (Signet; 1:15000) and anti-GFAP (DAKO; 1:2000) at RT. Antigens were visualized using Cy3- and DyLight488-labeled secondary antibodies (Jackson ImmunoResearch Laboratories and Invitrogen; 1:1400), incubated for 2 h at RT. Sections were washed and coverslipped in Vectashield including DAPI as a nuclear dye (Vector Laboratories). PV and WFA stainings were performed on free-floating brain sections obtained from animals at 3 months of age. Sections were quenched with 10% (v/v) methanol and 0.3% (v/v) H_2_O_2_ in PBS for 30 min at RT, blocked with 0.2% (v/v) Triton X-100 and 5% (v/v) fetal bovine serum in PBS, and incubated overnight with anti-PV (Swant, Marly, Switzerland; 1:1000) and fluorescein labeled WFA (Vector Laboratories; 1:400) at RT. PV staining was visualized using a Alex568-labeled secondary antibodies (Invitrogen; 1:400), incubated for 2 h at RT. Sections were washed and coverslipped in Vectashield including DAPI as a nuclear dye (Vector Laboratories). PV and WFA staining were quantified using ImageJ v1.48. PV- and WFA-positive cells were counted using image thresholding and automated particle analysis in ImageJ (v1.48).

### Intra-hippocampal injections

Mice were anesthetized with avertin (1.2% (w/v)), 0.02 ml/g, intraperitoneal) and chronically implanted with double guide cannulas in the CA1 region of the dorsal hippocampus as previously described [32]. Buprenorphine (0.1 mg/kg, subcutaneous) was injected as an analgesic. Mice were allowed to recover for 5 days before experimentation. Chondroitinase ABC or penicillinase (both from Sigma) was injected at 0.025 U/side 24 h before fear conditioning or 3–4 days before electrophysiological recording. In all injection experiments APP/PS1 transgenic mice and wildtype littermate controls were used at 3 months of age.

### Contextual fear conditioning

All experiments were carried out in a fear conditioning system (TSE Systems) as previously described [32]. Training and testing was performed in a Plexiglas chamber with a stainless steel grid floor with constant illumination (100–500 lx) and background sound (white noise, 68 dB sound pressure level), situated in a gray box to shield it from the outside. The chamber was cleaned with 70% ethanol before each session. Training consisted of placing mice in the chamber for a period of 180 s, after which a 2 s foot shock (0.7 mA) was delivered through the grid floor. Mice were returned to their home cage 30 s after the end of the shock. The retrieval tests consisted of a 180 s re-exposure to the context (conditioned stimulus) 24 h after acquisition. Baseline inactivity, exploration, distance traveled and freezing were assessed automatically. Freezing was defined as lack of any movement besides respiration and heart beat during 4 s intervals and is presented as a percentage of the total test time. Significance was determined using a Student *t*-test (untreated animals) or Two-Way ANOVA (penicillinase and chondroitinase ABC treated animals) followed by one-sided Student *t*-test *post-hoc* analysis.

### Morris water maze

Spatial memory was tested in a Morris water maze setup. Before testing, mice were handled for 5 days. A circular pool (ø 1.2 m) was filled with water which was painted white with non-toxic paint and kept at a temperature of 25°C. An escape platform (ø 9 cm) was placed at 30 cm from the edge of the pool submerged 0.5 cm below the water surface. Visual cues were located around the pool at a distance of ~1 m. During testing lights were dimmed and covered with white sheets and mice were video-tracked using Viewer (BiobServe, Fort Lee, NJ). Mice were trained for 5 consecutive days using 4 trials/day with a 30–180 s inter-trial interval. In each trial, mice were first placed on the platform for 30 s, and then placed in the water at a random start position and allowed a maximum of 60 s to find the platform. Mice that were unable to find the platform within 60 s were placed back on the platform by hand. After 30 s on the platform mice were tested again. To prevent hypothermia mice were placed in their home cage for 3 min between trials 2 and 3. On day 6 a probe trial was performed with the platform removed. Mice were placed in the pool opposite from the platform location and allowed to swim for 60 s. During training trials, the latency to reach the platform was measured; in the probe trial, the time spent in each quadrant of the pool was measured. Learning was assessed as the amount of time spent in the target quadrant. Significance was determined using a paired Student *t*-test (for learning within genotypes) or an unpaired Student *t*-test (for differences between genotypes).

### Long-term potentiation

A planar multi-electrode recording setup (MED64 system; Alpha Med Sciences, Tokyo, Japan) was used to record field excitatory post-synaptic potential (fEPSP) and to elicit LTP as previously described [33]. Animals were decapitated and brains were rapidly removed and placed in ice-cold slice buffer (124 mM NaCl, 3.3 mM KCl, 1.2 mM KH_2_PO_4_, 7 mM MgSO_4_, 0.5 mM CaCl_2_, 20 mM NaHCO_3_ and 10 mM glucose; constantly gassed with 95% O_2_/5% CO_2_). Coronal hippocampal slices were prepared using a vibrating microtome at 400 μm and then placed in a chamber containing artificial cerebrospinal fluid (aCSF; 124 mM NaCl, 3.3 mM KCl, 1.2 mM KH_2_PO_4_, 1.3 mM MgSO_4_, 2.5 mM CaCl_2_, 20 mM NaHCO_3_ and 10 mM glucose; constantly gassed with 95% O_2_/5% CO_2_). Slices were allowed to recover for 1 h and then placed on 8×8 multi-electrode arrays containing P5155 probes (Alpha Med Sciences; inter-electrode distance 150 μm) pre-coated with polyethylenimine (PEI; Sigma). After addition of 500 μl aCSF the array was placed in a moist chamber that was constantly gassed with 95% O_2_/5% CO_2_ for at least 1 h before recording. Correct placement of the electrodes at the CA3–CA1 region was done manually, monitored by a microscope (SZ61; Olympus, Japan). During recording, slices were constantly perfused with oxygenated aCSF containing 10 μM glycine at a flow rate of 2 mL/min at RT. fEPSPs were recorded from multiple electrodes in the stratum radiatum of CA1. An external concentric bipolar electrode (CBCBG75; FH-Company, Bowdoin, ME) in the Schaffer collateral pathway was used as the stimulating electrode using a homemade model 440b isolated Bipolar Current Stimulator. Based on the stimulus–response curve, a stimulation intensity was used that evoked fEPSPs with a magnitude of 50% of the maximum response (usually ~100 μA). After allowing a stable baseline of 10 min, LTP was evoked by a 2× 100 Hz stimulus of 1 sec each with a 15 sec interval and fEPSP responses were recorded for 1 h after the tetanus. LTP was expressed as the change in the slope of the fEPSP relative to baseline and averaged for multiple electrodes (usually 5) located in the stratum radiatum. Statistical significance was determined using a Two-Way ANOVA on the average fEPSP change from 10–20 min after LTP induction followed by Student *t*-test *post-hoc* analysis.

## Results

### Hippocampal memory impairments in pre-pathological APP/PS1mice

**Figure 1:**
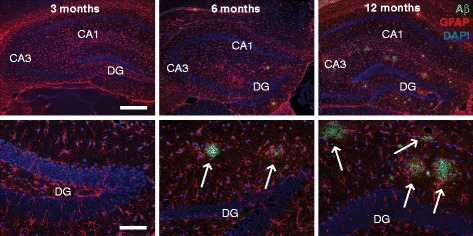
**Development of Aβ plaque pathology in the hippocampus of APP/PS1 mice.** No Aβ-positive (green) plaques are observed in the hippocampus of APP/PS1 mice at 3 months of age, whereas at 6 months, all mice had few hippocampal plaques (arrows). Plaque load is further increased at 12 months. Parallel to the increase in plaque formation, there was a moderate increase in GFAP staining (red) around all plaques at 6 and 12 months of age. CA, cornus ammonis; DG, dentate gyrus. Scale bars: upper panels, 400 μm; lower panels, 100 μm.

**Figure 2:**
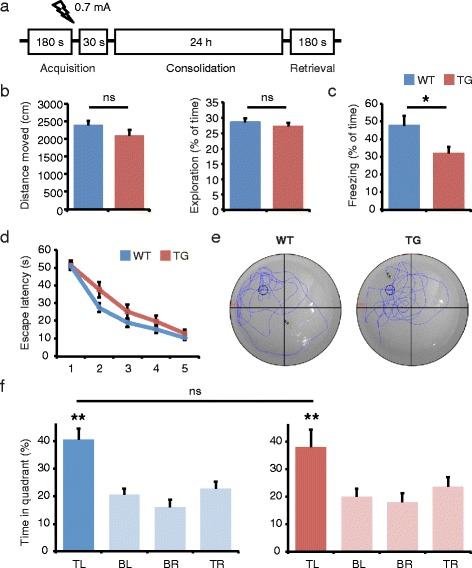
**Hippocampal memory performance is impaired in 3 months old APP/PS1 mice. a** Hippocampal memory performance at 3 months of age was tested in a contextual fear memory task. In the acquisition phase animals received a mild foot shock, and memory retrieval was tested 24 later by re-exposing animals to the shock-associated context. **b** APP/PS1 transgenic (TG) mice and wildtype (WT) littermates exhibited similar locomotor and exploratory activity during training trials before shock delivery. **c** Memory retrieval, as assessed by freezing behavior upon re-exposure of mice to the shock-associated context, was significantly reduced in APP/PS1 mice compared with wildtype mice; *n* = 10 (Student’s *t* test; mean ± SEM; ns, not significant; *p < 0.05). **d** Spatial memory was tested in a Morris water maze. During the 5-day training no differences were observed in escape latencies between APP/PS1 and wildtype mice. **e** Representative swim traces during the probe test show that APP/PS1 and wildtype mice both remembered the original location of the escape platform. **f** Quantification of quadrant occupancy shows that memory acquisition occurred equally in both genotypes, and that there was no significant difference in time spent in the top-left target quadrant. TL, top-left; TR, top-right; BL, bottom-left; BR, bottom-right; *n* = 10–13 (Student’s *t* test; mean ± SEM; ns, not significant; **p < 0.01).

We first determined the progression of AD-like pathology and memory impairments in APP/PS1 mice using immunohistochemistry and behavioral analysis. In accordance with previous reports [14,31], no Aβ plaques were observed at 3 months of age. At 6 months APP/PS1 mice had few hippocampal plaques, and at 12 months plaque load was further increased (Figure 1). We next evaluated hippocampal memory in 3 months old APP/PS1 mice that lack AD-like pathology. In a contextual fear-conditioning task (Figure 2a), APP/PS1 and wildtype littermates exhibited similar baseline activity during the acquisition phase before shock delivery (Figure 2b). Memory, however, was significantly reduced in APP/PS1 mice, as assessed by freezing behavior during the memory retrieval phase upon re-exposure of the animals to the context (Figure 2c). When we tested spatial memory performance in a Morris water maze task using a separate batch of animals, no differences were observed between APP/PS1 mice and wildtype littermates at 4 months of age; wildtype and transgenic animals performed equally well, both during the 5-day training (Figure 2d) and in the probe trial (Figure 2e-f). These data are in agreement with other studies showing that contextual fear memory is early affected in APP transgenic mouse models, whereas spatial and reference memory deficits only become apparent after 6–8 months [16,34,35]. We conclude that acquisition of contextual fear memory can be used as a robust and reliably measure of pre-pathological memory deficits in APP/PS1 mice.

### Increased hippocampal extracellular matrix protein levels and perineuronal net densities in pre-pathological APP/PS1 mice

We next asked what might be the molecular basis of early hippocampal memory impairments in APP/PS1 mice. Proteomics analysis was performed on hippocampal synaptosome fractions at 1.5 and 3 months of age, before plaque deposition, and at 6 and 12 months of age, after the onset of plaque formation (Additional file 2: Figure S1). A total of 376 proteins were quantified (Additional file 3: Table S1), of which the levels of 105 proteins were significantly different between APP/PS1 mice and wildtype controls at one or more time points (FDR <10, log-fold change >0.125). Most differentially regulated proteins, 87 in total, were detected at 3 months of age. The most differentially regulated protein detected was APP, showing significantly increased levels in APP/PS1 mice compared with wildtype controls at 3, 6 and 12 months of age, reaching a maximum of 2.5-fold increase at 12 months (Additional file 4: Figure S2a). Higher APP levels in APP/PS1 mice could to a large extent be attributed to an increase of the specific Aβ peptide LVFFAEDVGSNK, in particular at 12 months of age (Additional file 4: Figure S2b). This was confirmed by immunoblotting with an Aβ-specific antibody (Additional file 4: Figure S2c). These data demonstrate that APP/PS1 mice have an age-dependent accumulation of Aβ in hippocampal synaptosome fractions.

**Figure 3:**
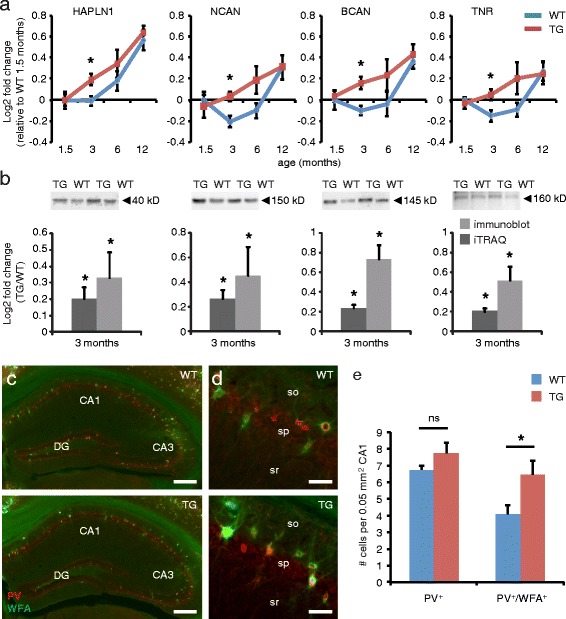
**Hippocampal ECM protein levels are increased in 3 months old APP/PS1 mice. a** Time course profiles of four ECM proteins, hyaluronan and proteoglycan link protein 1 (HAPLN1), neurocan (NCAN), brevican (BCAN) and tenascin-R (TNR), reveal significantly higher levels in APP/PS1 transgenic (TG) mice compared with wildtype (WT) littermates at 3 months of age; *n* = 5 mice per genotype (SAM analysis; mean ± SEM; *FDR < 10). **b** Higher ECM protein levels were confirmed by immunoblotting; *n* = 6 mice per genotype (Student’s *t* test; mean ± SEM; *p < 0.05). **c**,**d** PV/WFA double-labeling of coronal sections of the hippocampus confirms an increase in the number of WFA-positive PNNs around PV-positive cells in APP/PS1 transgenic (TG) mice compared with wildtype (WT) controls. CA, cornus ammonis; DG, dentate gyrus; sp, stratum pyramidale; sr, stratum radiatum; so, stratum oriens. Scale bars: 250 μm **(c)**, 40 μm **(d)**. **e** Quantification of PV-positive and PV/WFA-double-positive cells in the CA1 area reveals a significant increase in the number of PV-positive cells containing WFA-positive PNNs in APP/PS1 mice compared with wildtype controls, whereas the total number of PV-positive cells remained unaltered; *n* = 16 sections from 4 mice per genotype (Student’s *t* test; mean ± SEM; *p < 0.05).

Typical synapse organizing proteins, such as the postsynaptic protein PSD-95 and the presynaptic proteins bassoon and piccolo, were not significantly different between APP/PS1 mice and wildtype controls, neither were the major hippocampal AMPA- and NMDA-type glutamate receptors (Additional file 3: Table S1), suggesting that other synaptic alterations underlie the observed cognitive defects in APP/PS1 mice. Functional enrichment analysis revealed the strongest enrichment at 3 months of age for proteins of the extracellular matrix (ECM; Additional file 5: Table S2). Time course expression analysis of four ECM proteins, i.e., the chondroitin sulphate proteoglycans (CSPGs) neurocan and brevican, and the proteoglycan crosslinking proteins tenascin-R and hyaluronan/proteoglycan link protein 1, revealed a gradual age-dependent upregulation in wildtype mice, which was accelerated in APP/PS1 mice resulting in higher relative expression levels at 3 and 6 months of age (Figure 3a). The initial downregulation of neurocan in wildtype mice is in line with its proposed juvenile function [36]. The upregulation of ECM proteins at 12 months of age in both APP/PS1 and wildtype mice marks the onset of an age-dependent increase in ECM levels that we recently also observed in normal aging mice and which continues progressively up to two years of age [37]. The relatively high levels of ECM proteins in APP/PS1 mice compared with wildtype controls at 3 months of age were confirmed by immunoblotting (Figure 3b). APP/PS1 mice also showed increased hippocampal staining using the ECM marker *Wisteria floribunda* agglutinin (WFA) [38] (Figure 3c-d). In particular, we observed a significant increase in the number of parvalbumin (PV)-positive cells containing WFA-positive perineuronal nets (PNNs), whereas the total number of PV-positive cells did not increase (Figure 3e).

### Chondroitinase ABC treatment restores hippocampal memory performance in pre-pathological APP/PS1 mice

**Figure 4:**
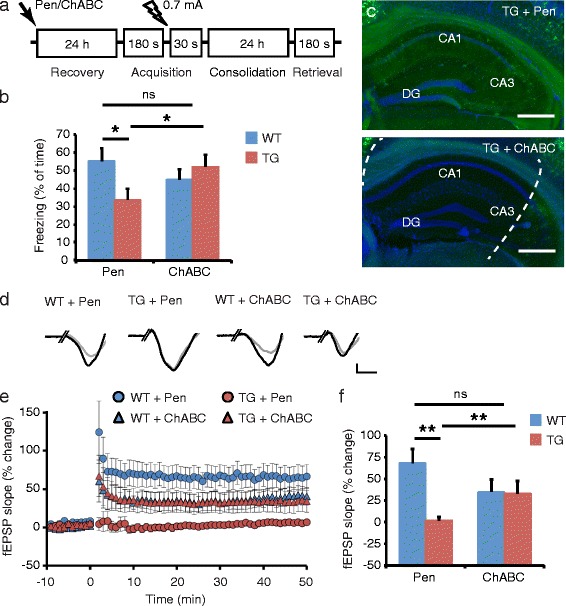
**ChABC treatment restores hippocampal memory and LTP in 3 months old APP/PS1 mice. a** Contextual fear memory was tested in animals that received intra-hippocampal injections of either penicillinase (Pen) or chondroitinase ABC (ChABC) 24 h prior to the test. **b** Memory retrieval was significantly reduced in Pen treated APP/PS1 transgenic (TG) mice (*n* = 10) compared with Pen treated wildtype (WT) mice (*n* = 8). ChABC treatment rescued memory performance in APP/PS1 mice (*n* = 7) but had no effect in wildtype mice (*n* = 8) (genotype x treatment Two-Way ANOVA: *F*_*1,30*_ = 4.41, *p* = 0.0442; mean ± SEM; ns, not significant; *p < 0.05). **c** WFA-staining confirmed a reduction in ECM levels due to injection of ChABC directly into the hippocampus. Dashed lines mark the hippocampal region that is affected by ChABC treatment. CA, cornus ammonis; DG, dentate gyrus. Scale bars: 400 μm. **d** LTP was induced in hippocampal slices obtained from Pen and ChABC treated APP/PS1 and wildtype mice. Single fEPSP traces before (grey) and after (black) tetanus stimulation are shown for each condition. Scale bars: 0.1 mV; 2 ms. **e** LTP was elicited in slices of Pen treated wildtype mice (*n* = 6), but not in slices of Pen treated APP/PS1 mice (*n* = 11). ChABC treatment partially rescued LTP in APP/PS1 mice (*n* = 7) but also slightly reduced LTP in wildtype mice (*n* = 3) (mean ± SEM). **f** Statistical analysis of the average fEPSP change at 10–20 min showed a significant LTP impairment in Pen treated APP/PS1 mice compared with Pen treated wildtype mice, and a significant LTP rescue in ChABC treated APP/PS1 mice compared with Pen treated APP/PS1 mice (genotype x treatment Two-Way ANOVA: *F*_*1,23*_ = 6.87, *p* = 0.0153; mean ± SEM; ns, not significant; **p < 0.01).

CSPGs are the major inhibitory components of both PNNs and diffuse ECM structures [39,40], and *in vivo* digestion of the chondroitin sulfate glycosaminoglycan side chains of CSPGs by chondroitinase ABC (ChABC) restores ocular dominance plasticity [41,42], promotes recovery from spinal cord injury [43], and enhances erasure of fear memory [44], object recognition learning and long-term depression [45], all without adverse side effects. We therefore asked whether local injection of ChABC (0.025 U/side) into the hippocampus of 3 months old APP/PS1 mice could reverse the observed fear memory deficit. Control animals were injected with penicillinase, an enzyme with no endogenous substrate in mice. Animals were subjected to the fear conditioning task 24 h after injection (Figure 4a). APP/PS1 mice that were injected with penicillinase showed a significant reduction in fear memory compared with wildtype penicillinase treated mice (Figure 4b), and this effect was comparable to untreated mice (Figure 2c). ChABC treatment on the other hand restored memory function in APP/PS1 mice, and freezing levels were not significantly different from either ChABC or penicillinase treated wildtype mice (Figure 4b). The efficacy and regional specificity of the ChABC treatment was confirmed by post-hoc staining of hippocampal sections with WFA (Figure 4c).

### Chondroitinase ABC treatment restores hippocampal long-term potentiation in pre-pathological APP/PS1 mice

At the cell physiological level contextual memory is represented as a long-term potentiation (LTP) of hippocampal synapses [46]. We therefore tested whether hippocampal LTP is affected in APP/PS1 mice and can be rescued by ChABC treatment. Hippocampal slices were prepared 24–48 h after injection of penicillinase or ChABC and LTP was induced by electrical stimulation of the Schaffer collateral pathway. Field excitatory postsynaptic potentials (fEPSP) were recorded in the stratum radiatum in CA1. Tetanic stimulation readily induced LTP in slices obtained from penicillinase treated wildtype mice, but LTP was significantly reduced in slices obtained from penicillinase treated APP/PS1 mice (Figure 4d-f). These findings are in accordance with previous LTP measurements in APP/PS1 mice [47,48]. ChABC treatment however significantly restored LTP in APP/PS1 mice, and LTP levels were not significantly different from either ChABC or penicillinase treated wildtype mice (Figure 4f). ChABC treatment thus rescues both behavioral and physiological deficits in APP/PS1 mice at 3 months of age.

## Discussion

The earliest clinical manifestation of AD is memory loss due to hippocampal synaptic dysfunction [13]. Employing an unbiased proteomics screen we demonstrate an early and significant upregulation in hippocampal synaptosome preparations of several ECM proteins. This upregulation coincides with an increase in synaptic Aβ levels, but precedes Aβ plaque pathology. Importantly, early memory and LTP deficits in APP/PS1 mice could be reversed by acute and local inactivation of the ECM using ChABC. Previous studies showed an upregulation of the ECM in late stage AD patients as well as in 10 months old APP/PS1 mice [49], and suggest a neuroprotective role for ECM structures in human AD brains [50]. Our observations suggest that the increase in ECM levels occurs earlier, and in addition to being protective also contributes to early memory and plasticity impairments in AD. Our focus on synaptosomal preparations may have contributed to the detection of localized ECM alterations that remained undetected in previous studies.

As a measure of hippocampal memory deficits we used contextual fear memory acquisition. We observed that 3–4 months old APP/PS1 mice are impaired in this task, but that spatial reference memory, as measured in a Morris water maze, is still intact. Impaired contextual fear memory at 3 months of age was previously reported for APP mice [16], although other studies suggest the absence of fear memory deficits until 6 months of age [51,52]. These discrepancies are most likely due to differences in genetic background or in the training protocols used. The absence of a spatial reference memory deficit is in line with previous studies showing that water maze learning in APP/PS1 mice is only affected from 6–8 months of age [34,35]. Although contextual fear conditioning and maze learning both critically depend on the processing of contextual information in the hippocampus, fear memory acquisition requires a single pairing of context and shock, whereas maze learning involves repeated exposure of the animal to the context. One explanation might be that hippocampal plasticity deficits in 3–4 months old APP/PS1 mice are masked by repeated stimulation, whereas they are revealed in situations where animals need to process a single stimulus and respond adequately immediately. Alternatively, fear learning might use different hippocampal circuitry and thus be differently organized and differentially affected in APP/PS1 mice. The fear memory deficit at 3 months of age was paralleled by a strong reduction in LTP induction. This is in line again with previous LTP measurements in APP/PS1 mice [47,48], although the extent of the LTP deficit differs per study and probably depends on the stimulation and recording conditions used. We conclude that both behavioral plasticity (fear conditioning) and physiological plasticity (LTP) are early affected in APP/PS1 mice, and that local degradation of the ECM with ChABC reverses these early deficits.

Interestingly, at 12 months of age, wildtype mice also show an increase in ECM protein levels, and no differences are observed anymore between APP/PS1 and wildtype animals. This is in accordance with our recent findings that an age-dependent increase in hippocampal ECM levels correlates with normal age-dependent cognitive decline [37]. Apparently, age-dependent hippocampal ECM accumulation is accelerated in APP/PS1 mice and contributes to early memory impairments, whereas later in the disease, other pathological mechanisms are responsible for further cognitive decline.

ECM in the brain is organized in different specialized structures. PNNs are mesh-like structures that surround the cell body and proximal dendrites of many neurons, whereas perisynaptic matrix is associated with individual synapses [53]. Our finding that ECM proteins are upregulated in synaptosomal preparations from APP/PS1 mice suggests an increase in perisynaptic ECM levels. Neuronal synthesis and synaptic release of ECM proteins might contribute to this upregulation [49,54]. The importance of perisynaptic ECM structures was demonstrated in hippocampal slice preparations where local treatment with ChABC enhanced spine motility without affecting PNNs [55]. It was demonstrated that perisynaptic matrix forms a physical barrier that restrict the lateral diffusion of AMPA receptors at postsynaptic sites [56,57], and that activity-dependent local degradation of perisynaptic matrix results in an integrin receptor-dependent increase in LTP [58,59]. Perisynaptic ECM could thus potentially contribute to the plasticity deficits observed in APP/PS1 mice. However, we also showed an increase in ECM-containing PNNs, in particular around PV interneurons. The number of PV neurons in CA1 with WFA-positive PNNs significantly increased from ~60% in wildtype mice to ~80% in APP/PS1 mice, whereas the total number of PV neurons remained unchanged. These findings suggest that PNNs at least also contribute to the observed memory impairments.

Previous studies reported that 50-60% of the PV neurons contain PNNs [60]. PNNs regulate diverse aspects of brain plasticity [61]. A developmental increase in cortical PNNs for instance corresponds with the ending of critical periods and the maturation of cortical circuits [62], and ChABC treatment can reactivate ocular dominance plasticity in the adult visual cortex [41,42]. In that respect it is interesting to note that APP/PS1 mice lack ocular dominance plasticity in the visual cortex at one month of age [63]. PNNs also regulate adult learning and memory. In the amygdala, PNNs protect fear memories from erasure, and local injection of ChABC into the amygdala enhances extinction of fear [44]. Genetic or ChABC-mediated degradation of PNNs in the perirhinal cortex enhances recognition memory [45], and injection of the ECM-degrading enzyme hyaluronidase into the auditory cortex of Mongolian gerbils promotes context-dependent auditory reversal learning [64]. A recent study showed for the first time that PV interneuron plasticity is also critically involved in the regulation of hippocampal learning and memory [65]. Learning was associated with a transient decrease in PV neuron activity, whereas a relatively high PV neuron activity was observed when memory was consolidated. ChABC treatment was able to induce a low activity state in PV neurons and enhanced learning, indicating the importance of PNNs in regulating hippocampal PV neuron activity. Interestingly, in AD patients, abnormal hippocampal network activity resulting from dysfunctional inhibitory interneurons is a well-established early pathological symptom [66].

At this moment we cannot distinguish the contribution of perisynaptic ECM from that of PNNs to the observed memory and plasticity deficits. Neither WFA staining nor ChABC treatment are specific enough to differentiate between these two possibilities, and likely they are both involved. In addition, it cannot be excluded that APP itself contributes to plasticity-restoring effects of ChABC. Shioi et al. [67] reported the existence of a chondroitin sulfate proteoglycan form of APP, which, if existent in APP/PS1 transgenic mice, could contribute to our observations. Future experiments should address these different possibilities using more sophisticated tools for ECM protein detection and intervention.

## Additional files

Additional file 1:
**Supplementary methods.**
Additional file 2: Figure S1. Schematic representation of the 8-plex iTRAQ approach. In each 8-plex iTRAQ experiment, combined left and right hippocampi of wildtype (WT) and transgenic (TG) mice of four different ages were labeled with one of the eight iTRAQ labels. After labeling the samples were pooled and subjected to MS/MS analysis for identification and quantification of proteins. In total, five replicate 8-plex experiments with independent biological samples were performed (*n* = 5). Additional file 3: Table S1. List of all quantified proteins. Additional file 4: Figure S2. APP/Aβ levels at hippocampal synaptic sites are significantly increased in APP/PS1 mice. **a** Proteomics analysis revealed a significant increase in APP levels at hippocampal synaptic sites in APP/PS1 transgenic (TG) mice compared with wildtype (WT) controls at 3, 6 and 12 months of age; *n* = 5 mice per genotype (SAM analysis; mean ± SEM; *FDR < 10). **b** Increased levels of APP as detected by proteomics analysis are primarily due to an increase in the levels of the Aβ-specific peptide LVFFAEDVGSNK (indicated in red), in particular at 12 months of age. **c** The increase in synaptic Aβ levels is further confirmed by immunoblotting using the Aβ-specific antibody 6E10. An age-dependent accumulation of monomeric and low molecular weight oligomeric Aβ is observed, starting as early as 1.5 months of age. Additional file 5: Table S2. Functional enrichment of differentially expressed proteins at 3 months of age. Significantly enriched (Fisher’s exact *p*-value <0.05) GO terms are listed. Enriched GO terms were assigned to two functional groups: extracellular matrix and presynaptic neurotransmitter release.
