# Supplementary material for: Reducing hippocampal extracellular matrix reverses early memory deficits in a mouse model of Alzheimer’s disease
Source: Acta Neuropathol Commun. 2014 Jun 29;2:76. doi: 10.1186/s40478-014-0076-z (PMC4149201; doi:10.1186/s40478-014-0076-z)
Supplement: Additional file 1: — Supplementary methods. [file 40478_2014_76_MOESM1_ESM.pdf]

Reducing hippocampal extracellular matrix reverses early memory deficits in a mouse model of Alzheimer's disease

Marlene J. Végh[a], Céline M. Heldring[a], Willem Kamphuis[b], Sara Hijazi[a], Arie J. Timmerman[a], Ka Wan Li[a], Pim van Nierop[a], Huibert D. Mansvelder[a], Elly M. Hol[c], August B. Smit[a], Ronald E. van Kesteren[a]

[a]Center for Neurogenomics and Cognitive Research, Neuroscience Campus Amsterdam, VU University, De Boelelaan 1085, 1081 HV Amsterdam, The Netherlands.

[b]Netherlands Institue for Neuroscience, Meibergdreef 47, 1105 BA Amsterdam, The Netherlands.

[c]Department of Translational Neuroscience, Brain Center Rudolf Magnus, University Medical Center Utrecht, Universiteitsweg 100, 3584 CG, Utrecht, The Netherlands.

Correspondence should be addressed to R.E. van Kesteren. E-mail: ronald.van.kesteren@vu.nl, phone: +31205987111, fax: +31205989281.

Supplementary Methods

Proteomics analysis

Sample preparation

Mouse hippocampi were dissected, frozen, and stored at -80°C until protein isolation. Synaptosomes were isolated from hippocampi of APP/PS1 mice and wildtype littermates at four different ages, 1.5, 3, 6 and 12 months of age as described previously [1,2] with minor modifications. In brief, hippocampi were homogenized in ice-cold 0.32 M sucrose buffer with 5 mM HEPES at pH 7.4 and protease inhibitor (Roche) and centrifuged at 1000 x *g* for 10 min at 4°C to remove debris. Supernatant was loaded on top of a discontinuous sucrose gradient consisting of 0.85 M and 1.2 M sucrose. After ultracentrifugation at 110,000 x *g* for 2 h at 4°C, the synaptosome fraction was collected at the interface of 0.85 M and 1.2 M sucrose, resuspended and pelleted by ultracentrifugation at 80,000 x *g* for 30 min at 4°C. After which the material was redissolved in 5 mM HEPES. Protein concentrations were determined using a Bradford assay (Bio-Rad, Hercules, CA). For each sample, 50 µg of protein was transferred to a fresh tube and dried in a SpeedVac overnight.

Protein digestion and iTRAQ labeling

Per sample, 50 µg synaptosome proteins were dissolved in 0.85% RapiGest (Waters Associates, Milford, MA), alkylated with methyl methanethiosulfonate, and digested with trypsin (sequencing grade; Promega, Madison, WI) as described [2,3]. In each iTRAQ experiment, four mutant samples were labeled respectively with iTRAQ reagents 113 (1.5 months), 114 (3 months), 115 (6 months), 116 (12 months), and four wildtype samples with iTRAQ reagents 117 (1.5 months), 118 (3 months), 119 (6 months), and 121 (12 months). To accommodate multiple independent biological replicates for each experimental condition (i.e., 2 genotypes x 4 time points), a total of five 8-plex iTRAQ experiments were performed.

Two-dimensional liquid chromatography (2DLC)

The lyophilized iTRAQ labeled samples were separated in the first dimension by strong cation exchange column (2.1x150 mm polysulfoethyl A column; PolyLC, Columbia, MD), and in the second dimension on an analytical capillary reverse phase C18 column (150 mm x 100 µm i.d. column) at 400 nL/min using the LC-Packing Ultimate system. The peptides were separated using a linear

gradient from 4% to 28% acetonitrile in 75 min, 28% to 36% in 7 min and finally to 72% in 2 min. The eluent was mixed with matrix (7 mg of re-crystallized α-cyano-hydroxycinnaminic acid in 1 ml 50% acetronitrile, 0.1% trifluoroacetic acid, 10 mM ammonium dicitrate) and delivered at a flow rate of 1.5 μL/min and deposited onto an Applied Biosystems matrix-assisted laser desorption ionization plated by means of a robot (Probot, Dionex; Thermo Scientific, Rockford, IL) once every 15 sec for a total of 384 spots.

MALDI-MS/MS

Samples were analyzed using an ABI 5800 proteomics analyzer (Applied Biosystems, Forster City, CA). Collision-induced dissociation of peptides was performed at 1 kV, the collision gas was air. MS/MS spectra were collected from 3000 laser shots. Peptides with signal-to-noise ratios over 50 at the MS mode were selected for MS/MS analysis, at a maximum of 20 MS/MS per spot. The precursor mass window was set to a relative resolution of 200.

Protein identification

Protein identification and quantification are described in detail in [4]. To annotate spectra, Mascot (MatrixScience, version 2.3.01) searches were performed against Swissprot (version 20/10/2010) and NCBInr (version 20/10/2010) databases. MS/MS spectra were searched with trypsin specificity and fixed iTRAQ modifications on lysine residues and N-termini of the peptides and methylthio modifications on cysteine residues. Oxidation on methionine residues was allowed as a variable modification. Mass tolerance was 200 ppm for precursor ions and 0.4 Da for fragment ions, while allowing a single site of miscleavage. The false discovery rate (FDR) for peptide identification was calculated using a randomized peptide database, and limited to include 5% false peptide discoveries. Protein redundancy of Swissprot and NCBInr searches was removed by clustering the precursor protein sequences at a threshold of 90% sequence similarity over 85% of the sequence length. Subsequently all peptides were matched against the protein clusters and only those peptides were included that mapped unique to one protein. Proteins were considered for quantification if at least three peptides were identified in three replicate iTRAQ sets and at least one peptide in all other sets.

Quantification of differentially expressed proteins

iTRAQ peak areas (m/z 113-121) were extracted. Peptides with an iTRAQ reporter ion peak intensity of 750 or lower were not considered for quantification. To compensate for small differences in the starting amounts of the samples, the individual peak areas of each iTRAQ signature peak were log transformed to yield a normal distribution, and normalized to the mean peak area per sample. Protein abundance was determined by taking the average normalized standardized iTRAQ peak area of all unique peptides annotated to that protein. Finally, the standardized protein means (four transgenic mice and four wildtype mice in each experiment) were used to calculate the average log-fold difference between WT and AD mice. Statistical significance was determined by calculating permutation-derived false discovery rates (*FDR*) using the SAM [5] package in MeV (version 4.6.1) [5,6]. Changes in protein expression are considered to be significant when the FDR is <10% and log-fold change >0.125. In addition, we have also provided the uncorrected *p*-value as determined by Student's *t*-test.

References

1 Klychnikov OI, Li KW, Sidorov IAet al. (2010) Quantitative cortical synapse proteomics of a transgenic migraine mouse model with mutated Ca(V)2.1 calcium channels. Proteomics 10: 2531-2535

2 Li KW, Miller S, Klychnikov Oet al. (2007) Quantitative proteomics and protein network analysis of hippocampal synapses of CaMKIIalpha mutant mice. J Prot Res 6: 3127-3133

3 Van den Oever MC, Goriounova NA, Li KWet al. (2008) Prefrontal cortex AMPA receptor plasticity is crucial for cue-induced relapse to heroin-seeking. Nat Neurosci 11: 1053-1058

4 van Nierop P, Loos M (2011) Bioinformatics procedures for analysis of quantitative proteomics experiments using iTRAQ. In: Li KW (ed) Neuroproteomics. Humana Press, City, pp 275-296

5 Tusher VG, Tibshirani R, Chu G (2001) Significance analysis of microarrays applied to the ionizing radiation response. Proc Natl Acad Sci U S A 98: 5116-5121

6 Saeed AI, Bhagabati NK, Braisted JCet al. (2006) TM4 microarray software suite. Methods Enzymol 411: 134-193
