# Supplementary material for: Reducing hippocampal extracellular matrix reverses early memory deficits in a mouse model of Alzheimer’s disease
Source: Acta Neuropathol Commun. 2014 Jun 29;2:76. doi: 10.1186/s40478-014-0076-z (PMC4149201; doi:10.1186/s40478-014-0076-z)
Supplement: Additional file 2: Figure S1. — Schematic representation of the 8-plex iTRAQ approach. In each 8-plex iTRAQ experiment, combined left and right hippocampi of wildtype (WT) and transgenic (TG) mice of four different ages were labeled with one of the eight iTRAQ labels. After labeling the samples were pooled and subjected to MS/MS analysis for identification and quantification of proteins. In total, five replicate 8-plex experiments with independent biological samples were performed (n = 5). [file 40478_2014_76_MOESM2_ESM.pdf]

Reducing hippocampal extracellular matrix reverses early memory deficits in a mouse model of Alzheimer's disease

Marlene J. Végh[a], Céline M. Heldring[a], Willem Kamphuis[b], Sara Hijazi[a], Arie J. Timmerman[a], Ka Wan Li[a], Pim van Nierop[a], Huibert D. Mansvelder[a], Elly M. Hol[c], August B. Smit[a], Ronald E. van Kesteren[a]

[a]Center for Neurogenomics and Cognitive Research, Neuroscience Campus Amsterdam, VU University, De Boelelaan 1085, 1081 HV Amsterdam, The Netherlands.

[b]Netherlands Institue for Neuroscience, Meibergdreef 47, 1105 BA Amsterdam, The Netherlands.

[c]Department of Translational Neuroscience, Brain Center Rudolf Magnus, University Medical Center Utrecht, Universiteitsweg 100, 3584 CG, Utrecht, The Netherlands.

Correspondence should be addressed to R.E. van Kesteren. E-mail: ronald.van.kesteren@vu.nl, phone: +31205987111, fax: +31205989281.

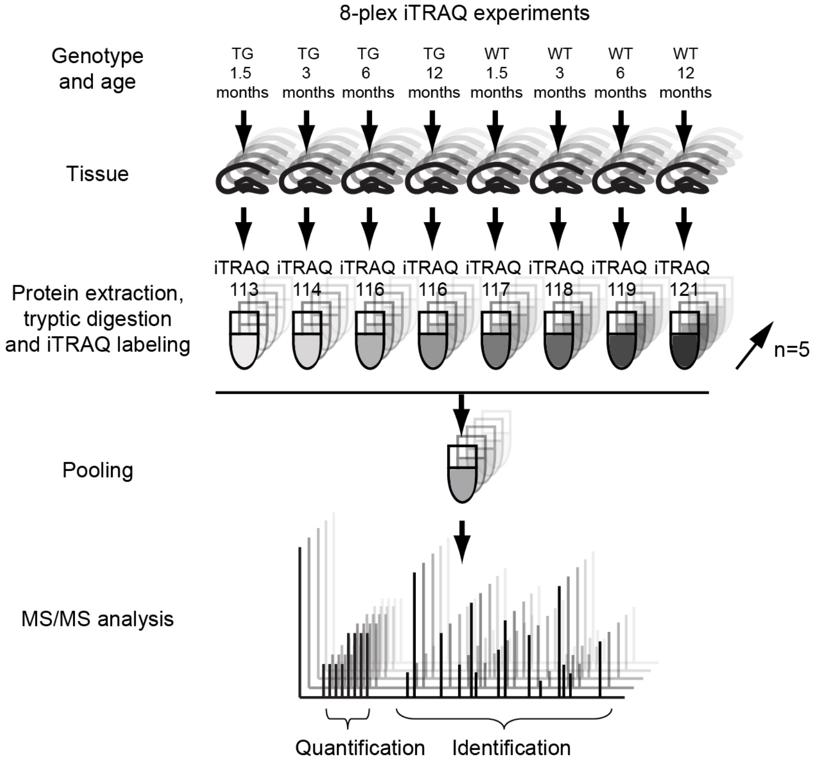

Suppl. Fig. 1 Schematic representation of the 8-plex iTRAQ approach. In each 8-plex iTRAQ experiment, combined left and right hippocampi of wildtype (WT) and transgenic (TG) mice of four different ages were labeled with one of the eight iTRAQ labels. After labeling the samples were pooled and subjected to MS/MS analysis for identification and quantification of proteins. In total, five replicate 8-plex experiments with independent biological samples were performed (*n* = 5)
