# Supplementary material for: Reducing hippocampal extracellular matrix reverses early memory deficits in a mouse model of Alzheimer’s disease
Source: Acta Neuropathol Commun. 2014 Jun 29;2:76. doi: 10.1186/s40478-014-0076-z (PMC4149201; doi:10.1186/s40478-014-0076-z)
Supplement: Additional file 3: Table S1. — List of all quantified proteins. [file 40478_2014_76_MOESM3_ESM.pdf]

Reducing hippocampal extracellular matrix reverses early memory deficits in a mouse model of Alzheimer's disease

Marlene J. Végh[a], Céline M. Heldring[a], Willem Kamphuis[b], Sara Hijazi[a], Arie J. Timmerman[a], Ka Wan Li[a], Pim van Nierop[a], Huibert D. Mansvelder[a], Elly M. Hol[c], August B. Smit[a], Ronald E. van Kesteren[a]

[a]Center for Neurogenomics and Cognitive Research, Neuroscience Campus Amsterdam, VU University, De Boelelaan 1085, 1081 HV Amsterdam, The Netherlands.

[b]Netherlands Institue for Neuroscience, Meibergdreef 47, 1105 BA Amsterdam, The Netherlands.

[c]Department of Translational Neuroscience, Brain Center Rudolf Magnus, University Medical Center Utrecht, Universiteitsweg 100, 3584 CG, Utrecht, The Netherlands.

Correspondence should be addressed to R.E. van Kesteren. E-mail: ronald.van.kesteren@vu.nl, phone: +31205987111, fax: +31205989281.

Supplementary Table 1

Suppl. Table 1 List of all quantified proteins

ID	Gene symbol	Description	Log-fold change 1.5 months TG:WT	Log-fold change 3 months TG:WT	Log-fold change 6 months TG:WT	Log-fold change 12 months TG:WT	STDEV 1.5 months	STDEV 3 months	STDEV 6 months	STDEV 12 months	SAM FDR % 1.5 months	SAM FDR % 3 months	SAM FDR % 6 months	SAM FDR % 12 months	*t*-test 1.5 months	*t*-test 3 months	*t*-test 6 months	*t*-test 12 months	Number of quantified peptides Set 1	Number of quantified peptides Set 2	Number of quantified peptides Set 3	Number of quantified peptides Set 4	Number of quantified peptides Set 5
THIL_MOUSE	ACAT1	Acetyl-CoA acetyltransferase	0.128	-0.024	0.007	-0.012	0.049	0.081	0.080	0.079	25.67	>100.00	84.08	>100.00	0.031	0.779	0.932	0.883	4	6	5	5	5
ACON_MOUSE	ACO2	Aconitate hydratase	0.020	-0.093	-0.131	0.045	0.041	0.072	0.159	0.087	73.92	40.16	54.74	52.60	0.635	0.246	0.501	0.621	12	15	14	12	14
ACSL6_MOUSE	ACSL6	Long-chain-fatty-acid--CoA ligase 6	0.078	0.020	0.078	-0.126	0.077	0.084	0.130	0.101	51.06	>100.00	77.57	56.85	0.360	0.821	0.580	0.252	2	7	6	4	2
ACTC_MOUSE	ACTC	Actin, alpha cardiac muscle 1	-0.107	-0.192	-0.117	0.152	0.092	0.071	0.243	0.176	53.37	5.18	70.10	42.52	0.278	0.044	0.632	0.419	13	13	10	11	11
ARP2_MOUSE	ACTR2	Actin-related protein 2	0.050	-0.080	0.033	-0.192	0.052	0.047	0.078	0.080	54.63	38.47	84.42	15.09	0.374	0.129	0.703	0.048	6	9	9	8	7
ARP3_MOUSE	ACTR3	Actin-related protein 3	0.036	-0.068	0.090	0.059	0.062	0.090	0.035	0.057	71.43	45.18	77.57	51.06	0.593	0.471	0.062	0.337	5	7	7	5	4
ADA23_MOUSE	ADAM23	ADAM 23	-0.186	0.220	0.065	-0.066	0.142	0.080	0.123	0.072	51.02	4.86	77.57	65.26	0.253	0.026	0.622	0.397	2	3	3	4	1
SAHH2_MOUSE	AHCYL1	Putative adenosylhomocysteinase 2	-0.185	0.042	-0.056	0.116	0.070	0.098	0.070	0.171	32.58	38.09	70.39	51.06	0.044	0.681	0.432	0.527	7	7	6	6	3
CD166_MOUSE	ALCAM	CD166 antigen	-0.050	-0.225	0.223	0.165	0.096	0.156	0.174	0.130	66.90	13.70	77.57	33.94	0.620	0.208	0.316	0.257	1	6	5	5	5
ALDOA_MOUSE	ALDOA	Fructose-bisphosphate aldolase A	-0.049	0.073	0.163	-0.114	0.071	0.058	0.094	0.095	66.90	45.18	77.57	59.68	0.514	0.246	0.115	0.272	15	16	13	14	15
ALDOC_MOUSE	ALDOC	Fructose-bisphosphate aldolase C	-0.072	-0.067	0.020	0.299	0.089	0.066	0.112	0.068	66.90	43.44	84.42	2.86	0.440	0.343	0.866	0.002	4	5	4	6	4
AMPH_MOUSE	AMPH	Amphiphysin	-0.038	-0.234	-0.229	-0.079	0.058	0.064	0.217	0.149	66.90	0.00	48.33	35.25	0.543	0.017	0.330	0.609	6	8	8	9	9
148680322	ANK2	Ankyrin 2, brain, isoform CRA_b	0.006	-0.023	0.036	-0.021	0.038	0.039	0.062	0.052	76.98	>100.00	82.66	>100.00	0.876	0.566	0.632	0.695	11	30	24	25	15
ANS1B_MOUSE	ANKS1B	Ankyrin repeat and sterile alpha motif domain-containing protein 1B	-0.001	0.098	0.057	-0.079	0.041	0.096	0.149	0.043	>100.00	22.38	81.64	61.12	0.988	0.340	0.686	0.103	7	7	10	5	8
ANXA5_MOUSE	ANXA5	Annexin A5	-0.121	0.213	-0.112	0.142	0.055	0.091	0.184	0.120	36.04	6.84	65.82	37.40	0.070	0.047	0.548	0.299	3	3	3	4	1
ANXA6_MOUSE	ANXA6	Annexin A6	-0.039	0.152	0.055	0.107	0.047	0.024	0.073	0.043	66.90	3.14	78.68	33.94	0.429	0.001	0.480	0.040	11	18	21	19	10
ANXA7_MOUSE	ANXA7	Annexin A7	0.107	0.008	0.014	0.056	0.108	0.093	0.117	0.056	50.52	>100.00	84.42	51.06	0.358	0.935	0.903	0.358	3	5	2	4	1
AP2A1_MOUSE	AP2A1	AP-2 complex subunit alpha-1	0.078	-0.061	-0.003	-0.043	0.050	0.046	0.059	0.081	38.56	42.52	>100.00	>100.00	0.162	0.221	0.966	0.612	7	16	13	15	11
AP2A2_MOUSE	AP2A2	AP-2 complex subunit alpha-2	0.032	-0.012	-0.050	0.090	0.041	0.052	0.068	0.049	66.90	>100.00	70.13	42.52	0.465	0.831	0.520	0.107	8	13	13	12	13
AP2B1_MOUSE	AP2B1	AP-2 complex subunit beta-1	0.122	0.027	-0.149	0.148	0.029	0.058	0.118	0.058	13.12	40.16	48.33	27.41	0.005	0.657	0.228	0.050	6	7	6	6	5
AP2M1_MOUSE	AP2M1	AP-2 complex subunit mu-1	0.000	0.005	-0.017	0.143	0.058	0.061	0.055	0.058	>100.00	>100.00	82.61	29.86	0.996	0.937	0.757	0.039	9	14	11	12	10
AP2S1_MOUSE	AP2S1	AP-2 complex subunit sigma-1	-0.090	0.139	0.071	0.257	0.066	0.070	0.124	0.082	51.02	13.62	77.57	17.68	0.224	0.084	0.585	0.015	3	5	4	5	4
APOE_MOUSE	APOE	Apolipoprotein E	-0.074	0.192	0.138	0.737	0.103	0.105	0.070	0.140	66.90	13.60	77.57	0.00	0.505	0.114	0.127	0.001	1	3	6	4	3
A4_MOUSE	APP	Amyloid beta A4 protein	0.183	0.386	0.408	1.325	0.120	0.131	0.083	0.265	32.80	3.50	0.00	0.00	0.167	0.019	0.004	0.003	2	3	5	5	2
AQP4_MOUSE	AQP4	Aquaporin-4	0.062	0.107	0.052	0.360	0.084	0.092	0.177	0.138	59.24	21.11	81.76	11.39	0.481	0.278	0.763	0.043	4	3	3	3	3
ARF3_MOUSE	ARF3	ADP-ribosylation factor 3	0.155	-0.146	-0.270	0.330	0.087	0.070	0.266	0.187	26.24	9.59	48.33	21.15	0.142	0.071	0.357	0.118	3	4	3	3	2
ARC1A_MOUSE	ARPC1A	Actin-related protein 2/3 complex subunit 1A	-0.170	0.120	-0.084	-0.010	0.102	0.103	0.098	0.115	42.55	21.11	70.88	>100.00	0.160	0.278	0.389	0.931	1	5	4	4	2
ARPC2_MOUSE	ARPC2	Actin-related protein 2/3 complex subunit 2	-0.026	0.010	-0.005	-0.062	0.071	0.061	0.095	0.053	69.52	>100.00	>100.00	65.26	0.723	0.876	0.960	0.286	6	10	10	9	9
ARPC3_MOUSE	ARPC3	Actin-related protein 2/3 complex subunit 3	-0.063	0.104	-0.046	-0.191	0.063	0.072	0.160	0.154	62.80	19.86	73.06	44.46	0.346	0.186	0.792	0.251	2	4	5	3	3
ARPC4_MOUSE	ARPC4	Actin-related protein 2/3 complex subunit 4	0.017	0.081	0.020	-0.074	0.082	0.068	0.091	0.127	76.98	22.38	84.42	65.26	0.841	0.278	0.845	0.581	5	6	6	2	3
ARP5L_MOUSE	Arpc5l	Actin-related protein 2/3 complex subunit 5-like protein	-0.042	-0.182	-0.062	-0.033	0.063	0.093	0.141	0.151	66.90	9.19	70.02	>100.00	0.535	0.085	0.680	0.832	2	3	4	5	1
AT1A1_MOUSE	ATP1A1	Sodium/potassium-transporting ATPase subunit alpha-1	-0.031	-0.030	0.033	-0.085	0.056	0.065	0.106	0.058	66.90	28.43	84.42	61.47	0.592	0.655	0.791	0.177	23	26	23	24	21
AT1A2_MOUSE	ATP1A2	Sodium/potassium-transporting ATPase subunit alpha-2	-0.002	0.015	0.144	-0.003	0.075	0.040	0.100	0.069	50.18	>100.00	77.57	>100.00	0.977	0.721	0.179	0.968	25	28	27	29	27
AT1A3_MOUSE	ATP1A3	Sodium/potassium-transporting ATPase subunit alpha-3	-0.019	0.021	0.130	0.017	0.063	0.043	0.106	0.056	69.52	40.16	77.57	>100.00	0.774	0.639	0.300	0.768	28	31	30	31	31
AT1B1_MOUSE	ATP1B1	Sodium/potassium-transporting ATPase subunit beta-1	-0.046	0.150	0.207	0.093	0.038	0.049	0.100	0.077	61.19	7.45	77.57	42.52	0.271	0.020	0.108	0.260	13	17	16	16	15
AT1B2_MOUSE	ATP1B2	Sodium/potassium-transporting ATPase subunit beta-2	-0.075	-0.071	0.144	-0.042	0.058	0.035	0.130	0.078	53.37	38.86	77.57	>100.00	0.235	0.081	0.318	0.601	7	7	7	7	5
AT2A2_MOUSE	ATP2A2	Sarcoplasmic/endoplasmic reticulum calcium ATPase 2	0.032	0.065	-0.032	0.045	0.023	0.024	0.043	0.037	52.48	21.11	75.29	51.06	0.213	0.026	0.502	0.263	9	14	14	15	8
AT2B2_MOUSE	ATP2B2	Plasma membrane calcium-transporting ATPase 2	-0.033	0.047	0.125	-0.059	0.054	0.040	0.106	0.055	66.90	31.23	77.57	65.26	0.562	0.279	0.310	0.321	10	19	16	15	13
148697948	ATP2B3	ATPase, Ca++ transporting, plasma membrane 3, isoform CRA_a	-0.011	0.106	0.108	-0.096	0.058	0.034	0.081	0.045	69.52	13.24	77.57	56.85	0.860	0.014	0.259	0.064	8	14	13	14	10
ATPA_MOUSE	ATP5A1	ATP synthase subunit alpha	0.198	0.144	-0.190	-0.044	0.096	0.072	0.138	0.104	25.67	14.02	48.33	>100.00	0.074	0.103	0.275	0.692	22	27	27	28	22
ATPB_MOUSE	ATP5B	ATP synthase subunit beta	0.174	0.138	-0.153	0.095	0.108	0.092	0.132	0.121	31.49	19.86	48.33	51.06	0.149	0.186	0.344	0.464	16	18	17	17	17
ATPG_MOUSE	ATP5C1	ATP synthase subunit gamma	0.187	-0.099	-0.147	-0.100	0.082	0.098	0.088	0.127	25.67	41.72	48.33	65.26	0.055	0.347	0.174	0.459	6	7	8	9	7
ATPD_MOUSE	ATP5D	ATP synthase subunit delta	0.217	0.152	-0.181	-0.019	0.153	0.129	0.086	0.161	35.78	19.86	48.33	>100.00	0.199	0.283	0.113	0.908	2	3	2	3	3
ATP5E_MOUSE	ATP5E	ATP synthase subunit epsilon	0.094	0.132	-0.324	0.096	0.134	0.163	0.150	0.185	59.24	23.67	48.33	51.06	0.506	0.461	0.098	0.629	2	3	3	3	1
AT5F1_MOUSE	ATP5F1	ATP synthase subunit b	0.205	0.040	-0.149	0.093	0.106	0.096	0.139	0.163	25.67	38.09	54.74	51.06	0.091	0.696	0.368	0.592	5	7	5	6	6
ATP5H_MOUSE	ATP5H	ATP synthase subunit d	0.186	0.011	-0.173	0.097	0.099	0.096	0.145	0.153	25.67	>100.00	48.33	51.06	0.100	0.913	0.303	0.542	3	10	6	9	8
ATP5I_MOUSE	ATP5I	ATP synthase subunit e	0.167	0.128	-0.224	0.177	0.139	0.104	0.131	0.143	38.56	19.86	48.33	33.94	0.279	0.253	0.208	0.254	2	4	3	5	4
ATP5J_MOUSE	ATP5J	ATP synthase-coupling factor 6	0.118	0.144	-0.173	0.180	0.149	0.098	0.095	0.185	54.63	19.86	48.33	37.40	0.455	0.191	0.181	0.364	1	4	4	3	3
ATP5L_MOUSE	ATP5L	ATP synthase subunit g	0.138	0.144	-0.151	0.102	0.153	0.101	0.189	0.139	51.06	19.86	54.74	51.06	0.395	0.198	0.513	0.492	3	5	4	4	3
ATPO_MOUSE	ATP5O	ATP synthase subunit O	0.106	0.100	-0.205	0.035	0.076	0.086	0.104	0.107	38.56	21.11	48.33	55.14	0.202	0.298	0.144	0.751	8	10	10	9	8
VPP1_MOUSE	ATP6V0A1	V-type proton ATPase 116 kDa subunit a isoform 1	-0.073	-0.006	0.082	0.021	0.045	0.036	0.077	0.051	51.02	>100.00	77.57	57.99	0.140	0.874	0.317	0.699	16	22	20	24	21
VA0D1_MOUSE	ATP6V0D1	V-type proton ATPase subunit d 1	-0.030	0.106	0.098	0.036	0.040	0.049	0.043	0.049	66.90	19.86	77.57	52.60	0.478	0.071	0.063	0.490	10	8	10	9	9
VATA_MOUSE	ATP6V1A	V-type proton ATPase catalytic subunit A	0.005	-0.194	-0.136	-0.085	0.064	0.050	0.129	0.121	>100.00	2.48	54.74	65.26	0.938	0.006	0.360	0.506	15	19	20	16	16
VATB2_MOUSE	ATP6V1B2	V-type proton ATPase subunit B, brain isoform	-0.062	-0.045	-0.036	0.015	0.068	0.041	0.145	0.133	63.05	45.18	75.29	>100.00	0.401	0.307	0.796	0.914	7	11	10	9	10
VATC1_MOUSE	ATP6V1C1	V-type proton ATPase subunit C 1	-0.024	-0.010	-0.023	0.024	0.046	0.048	0.098	0.079	67.56	>100.00	80.37	57.99	0.617	0.848	0.823	0.771	8	11	9	12	13
VATD_MOUSE	ATP6V1D	V-type proton ATPase subunit D	-0.047	-0.188	-0.021	0.180	0.158	0.121	0.155	0.094	69.52	14.02	82.60	27.41	0.777	0.175	0.901	0.095	1	5	5	4	5
VATE1_MOUSE	ATP6V1E1	V-type proton ATPase subunit E 1	-0.039	-0.208	-0.063	0.024	0.075	0.097	0.142	0.073	66.90	7.57	70.39	57.99	0.618	0.088	0.672	0.755	7	11	10	10	8
VATF_MOUSE	ATP6V1F	V-type proton ATPase subunit F	-0.027	0.008	-0.163	0.094	0.088	0.076	0.082	0.136	69.52	>100.00	48.33	51.06	0.766	0.916	0.093	0.511	3	4	3	4	3
VATG2_MOUSE	ATP6V1G2	V-type proton ATPase subunit G 2	-0.100	0.034	-0.080	0.097	0.035	0.042	0.138	0.114	32.80	35.97	71.00	51.06	0.042	0.437	0.585	0.425	5	5	5	5	5
VATH_MOUSE	ATP6V1H	V-type proton ATPase subunit H	-0.116	-0.243	-0.093	0.136	0.084	0.100	0.234	0.162	51.02	3.40	71.00	42.52	0.215	0.061	0.696	0.427	2	7	7	5	4
AT8A1_MOUSE	ATP8A1	Probable phospholipid-transporting ATPase IA	-0.021	0.101	0.021	-0.225	0.058	0.066	0.105	0.099	69.52	19.86	84.42	11.02	0.725	0.171	0.859	0.055	3	3	2	3	4
BAIP2_MOUSE	BAIAP2	Brain-specific angiogenesis inhibitor 1-associated protein 2	-0.077	0.015	0.047	-0.008	0.045	0.049	0.096	0.074	51.02	>100.00	81.76	>100.00	0.152	0.765	0.624	0.917	7	15	9	12	8
BASP_MOUSE	BASP1	Brain acid soluble protein 1	-0.188	-0.337	-0.193	0.290	0.130	0.075	0.292	0.232	51.02	0.00	54.74	29.86	0.187	0.009	0.515	0.247	12	12	11	12	12
PGCB_MOUSE	BCAN	Brevican core protein	-0.059	0.240	0.290	0.004	0.135	0.073	0.163	0.164	67.56	3.14	77.57	>100.00	0.675	0.012	0.102	0.980	6	8	8	7	8
BDH_MOUSE	BDH1	D-beta-hydroxybutyrate dehydrogenase	0.188	0.118	-0.026	0.088	0.116	0.100	0.124	0.144	30.45	21.11	80.37	51.06	0.151	0.285	0.852	0.559	5	7	8	6	6
BSN_MOUSE	BSN	Protein bassoon	0.025	0.041	0.131	-0.007	0.030	0.051	0.098	0.053	68.19	35.33	77.57	>100.00	0.434	0.450	0.232	0.900	21	56	46	48	30
CA2D1_MOUSE	CACNA2D1	Voltage-dependent calcium channel subunit alpha-2/delta-1	0.030	0.049	0.114	0.046	0.059	0.051	0.084	0.070	74.06	33.34	77.57	51.06	0.630	0.381	0.231	0.530	9	16	14	14	6
CA2D3_MOUSE	CACNA2D3	Voltage-dependent calcium channel subunit alpha-2/delta-3	-0.028	0.219	0.137	0.012	0.123	0.096	0.103	0.108	69.52	6.84	77.57	>100.00	0.824	0.064	0.279	0.915	1	3	3	4	2
CACB4_MOUSE	CACNB4	Voltage-dependent L-type calcium channel subunit beta-4	-0.133	0.316	-0.064	0.046	0.089	0.093	0.149	0.095	51.02	3.50	70.39	52.60	0.193	0.021	0.661	0.646	1	5	5	4	3
CCG8_MOUSE	CACNG8	Voltage-dependent calcium channel gamma-8 subunit	0.092	0.251	0.071	0.020	0.065	0.075	0.159	0.103	38.56	3.31	77.57	>100.00	0.195	0.012	0.655	0.853	4	6	7	7	2
CADM2_MOUSE	CADM2	Cell adhesion molecule 2	-0.054	0.043	0.219	0.058	0.080	0.060	0.106	0.116	66.90	35.33	77.57	51.06	0.527	0.494	0.071	0.636	4	6	4	3	5
CAPS1_MOUSE	CADPS	Calcium-dependent secretion activator 1	0.010	-0.081	-0.401	0.082	0.088	0.084	0.298	0.177	76.98	42.52	48.33	51.06	0.914	0.362	0.276	0.656	1	4	3	5	4
CALM_MOUSE	CALM1	Calmodulin	-0.108	-0.131	0.076	-0.001	0.091	0.111	0.108	0.194	53.37	35.97	77.57	>100.00	0.289	0.284	0.550	0.995	5	8	6	7	4
CALR_MOUSE	CALR	Calreticulin	-0.088	-0.007	-0.062	0.044	0.041	0.056	0.090	0.094	42.55	>100.00	70.39	52.60	0.072	0.909	0.496	0.661	4	7	4	9	8
KCC2A_MOUSE	CAMK2A	Calcium/calmodulin-dependent protein kinase type II alpha chain	-0.231	-0.323	0.080	-0.279	0.109	0.173	0.137	0.150	32.42	4.67	77.57	11.02	0.071	0.114	0.594	0.114	12	14	10	10	9
KCC2B_MOUSE	CAMK2B	Calcium/calmodulin-dependent protein kinase type II beta chain	-0.136	-0.450	0.127	-0.430	0.123	0.155	0.096	0.163	53.37	0.00	77.57	0.00	0.310	0.024	0.297	0.030	6	9	7	7	5
CAMKV_MOUSE	CAMKV	CaM kinase-like vesicle-associated protein	0.071	0.109	0.094	0.093	0.059	0.067	0.059	0.068	47.63	19.86	77.57	42.52	0.270	0.154	0.227	0.236	10	13	14	15	12
CALX_MOUSE	CANX	Calnexin	0.034	-0.116	0.043	-0.293	0.049	0.073	0.086	0.089	68.37	28.57	81.76	3.67	0.516	0.150	0.663	0.012	7	12	8	9	8
CAPZB_MOUSE	CAPZB	F-actin-capping protein subunit beta	-0.145	-0.097	0.018	0.069	0.090	0.104	0.151	0.099	48.32	42.46	84.42	51.06	0.145	0.380	0.913	0.511	3	2	3	3	2
OX2G_MOUSE	CD200	OX-2 membrane glycoprotein	-0.004	-0.134	-0.060	-0.279	0.090	0.058	0.094	0.084	>100.00	9.59	70.39	3.35	0.969	0.048	0.515	0.010	2	4	3	3	2

CDC42_MOUSE	CDC42	Cell division control protein 42 homolog	-0.060	-0.046	-0.035	0.144	0.095	0.043	0.127	0.085	66.90	45.18	75.29	33.94	0.548	0.321	0.794	0.131	3	4	4	4	3
CADH2_MOUSE	CDH2	Cadherin-2	0.091	-0.069	0.180	-0.065	0.109	0.119	0.112	0.093	54.63	46.49	77.57	65.26	0.429	0.585	0.200	0.507	1	5	2	3	3
CEND_MOUSE	CEND1	Cell cycle exit and neuronal differentiation protein 1	0.086	0.052	-0.178	0.193	0.109	0.091	0.122	0.191	56.97	35.33	48.33	35.22	0.452	0.583	0.265	0.349	5	6	6	8	5
COF1_MOUSE	CFL1	Cofilin-1	-0.028	-0.165	-0.332	0.181	0.088	0.077	0.203	0.167	69.52	9.19	48.33	33.94	0.763	0.064	0.131	0.308	6	12	5	7	6
CISD1_MOUSE	CISD1	CDGSH iron sulfur domain-containing protein 1	0.202	0.016	-0.068	-0.247	0.125	0.074	0.106	0.145	30.45	>100.00	69.92	18.10	0.145	0.833	0.546	0.130	3	5	5	4	2
KCRB_MOUSE	CKB	Creatine kinase B-type	-0.109	-0.215	-0.268	0.098	0.082	0.060	0.235	0.188	51.02	0.00	48.33	51.06	0.235	0.009	0.272	0.615	8	14	9	11	6
KCRU_MOUSE	CKMT1	Creatine kinase, ubiquitous mitochondrial	0.159	0.018	-0.131	0.115	0.078	0.078	0.084	0.092	25.67	>100.00	54.74	42.52	0.081	0.822	0.218	0.274	7	10	9	11	9
CLCB_MOUSE	CLTB	Clathrin light chain B	-0.110	-0.160	-0.236	0.139	0.132	0.068	0.162	0.186	62.80	9.19	48.33	45.40	0.445	0.047	0.178	0.478	4	4	3	2	3
CLH_MOUSE	CLTC	Clathrin heavy chain 1	-0.049	-0.341	-0.152	-0.120	0.098	0.071	0.171	0.158	66.90	0.00	54.74	63.47	0.633	0.005	0.392	0.469	32	41	32	37	34
CN37_MOUSE	CNP	2',3'-cyclic-nucleotide 3'-phosphodiesterase	-0.110	0.038	0.134	0.191	0.038	0.095	0.166	0.108	32.80	38.47	77.57	27.41	0.020	0.709	0.423	0.125	19	23	21	21	20
CNTN1_MOUSE	CNTN1	Contactin-1	-0.047	0.077	0.197	-0.119	0.062	0.045	0.115	0.069	66.90	21.11	77.57	56.85	0.472	0.140	0.190	0.130	19	30	25	23	19
CNTP1_MOUSE	CNTNAP1	Contactin-associated protein 1	0.027	0.203	0.098	0.014	0.058	0.064	0.043	0.052	73.92	4.06	77.57	33.94	0.661	0.014	0.049	0.796	7	14	14	15	7
COX41_MOUSE	COX4I1	Cytochrome c oxidase subunit 4 isoform 1	0.137	0.075	-0.269	-0.206	0.110	0.084	0.126	0.155	38.56	28.56	48.33	7.01	0.255	0.408	0.114	0.238	5	5	6	8	8
COX5A_MOUSE	COX5A	Cytochrome c oxidase subunit 5A	0.040	0.166	0.072	-0.155	0.112	0.103	0.127	0.123	75.99	15.16	77.57	56.85	0.730	0.146	0.610	0.251	3	6	6	7	5
COX5B_MOUSE	COX5B	Cytochrome c oxidase subunit 5B	0.181	0.139	-0.110	0.075	0.115	0.063	0.098	0.155	33.15	13.24	58.44	51.06	0.153	0.061	0.319	0.640	5	6	5	6	5
CX6B1_MOUSE	COX6B1	Cytochrome c oxidase subunit VIb isoform 1	0.216	-0.050	-0.246	-0.153	0.110	0.123	0.136	0.116	25.67	>100.00	48.33	56.85	0.087	0.698	0.108	0.223	5	6	6	5	7
COX6C_MOUSE	COX6C	Cytochrome c oxidase polypeptide VIc	0.170	0.120	-0.210	-0.114	0.097	0.074	0.141	0.140	26.24	19.86	48.33	63.47	0.118	0.160	0.250	0.456	4	8	4	7	8
CPNE4_MOUSE	CPNE4	Copine-4	0.022	0.071	-0.048	0.036	0.109	0.085	0.172	0.088	76.98	30.09	73.06	54.22	0.850	0.437	0.783	0.694	1	3	2	3	4
CPNE6_MOUSE	CPNE6	Copine-6	-0.003	0.120	0.080	0.093	0.055	0.040	0.074	0.054	>100.00	13.60	77.57	27.41	0.962	0.019	0.346	0.124	10	14	12	13	11
DPYL1_MOUSE	CRMP1	Dihydropyrimidinase-related protein 1	0.127	-0.239	0.018	0.105	0.151	0.089	0.247	0.220	53.37	2.29	84.42	51.06	0.426	0.036	0.947	0.646	5	4	1	4	2
CISY_MOUSE	CS	Citrate synthase	0.080	0.028	-0.108	0.053	0.049	0.054	0.093	0.057	35.78	38.47	58.44	51.06	0.140	0.624	0.361	0.401	11	11	11	10	9
CSRP1_MOUSE	CSRP1	Cysteine and glycine-rich protein 1	0.159	0.073	-0.148	0.237	0.117	0.103	0.185	0.163	38.56	31.23	54.74	27.41	0.215	0.498	0.432	0.191	1	4	2	4	3
CTNA2_MOUSE	CTNNA2	Catenin alpha-2	-0.044	0.050	0.071	0.007	0.043	0.052	0.063	0.043	62.80	31.23	77.57	>100.00	0.335	0.359	0.271	0.870	8	17	14	16	11
CTNB1_MOUSE	CTNNB1	Catenin beta-1	-0.062	-0.110	0.123	-0.254	0.034	0.065	0.140	0.048	51.02	28.57	77.57	0.00	0.110	0.141	0.425	0.001	9	18	15	16	11
CTND2_MOUSE	CTNND2	Catenin delta-2	-0.073	0.045	0.071	-0.168	0.112	0.089	0.053	0.094	66.90	36.56	77.57	30.51	0.541	0.630	0.198	0.120	1	6	9	6	4
CY1_MOUSE	CYC1	Cytochrome c1, heme protein	0.395	0.209	-0.177	-0.110	0.118	0.176	0.210	0.158	13.12	19.86	48.33	65.26	0.012	0.272	0.458	0.514	1	8	4	4	4
CYC_MOUSE	CYCS	Cytochrome c, somatic	-0.045	0.183	-0.107	0.068	0.089	0.060	0.149	0.065	66.90	5.52	66.03	65.26	0.627	0.016	0.469	0.328	5	7	6	6	5
CYFP2_MOUSE	CYFIP2	Cytoplasmic FMR1-interacting protein 2	-0.097	0.097	-0.047	0.070	0.077	0.077	0.075	0.073	53.37	21.11	70.13	65.26	0.244	0.242	0.554	0.365	3	7	4	6	4
DREB_MOUSE	DBN1	Drebrin	-0.094	0.029	-0.095	0.157	0.080	0.090	0.140	0.120	53.37	40.45	70.88	33.94	0.285	0.757	0.514	0.227	2	6	4	1	3
ODP2_MOUSE	DLAT	Dihydrolipoyllysine-residue acetyltransferase component of pyruvate dehydrogenase complex	0.042	0.013	-0.048	0.104	0.076	0.073	0.152	0.080	71.43	>100.00	73.06	42.52	0.599	0.865	0.780	0.234	4	7	4	4	4
DLG2_MOUSE	DLG2	Disks large homolog 2	-0.013	0.002	0.109	-0.079	0.055	0.048	0.104	0.082	69.52	>100.00	77.57	65.26	0.817	0.965	0.297	0.367	5	12	7	5	7
DLG3_MOUSE	DLG3	Disks large homolog 3	-0.065	0.140	0.208	0.044	0.031	0.050	0.144	0.057	50.18	9.08	77.57	51.06	0.090	0.026	0.234	0.467	2	6	5	7	6
DLG4_MOUSE	DLG4	Disks large homolog 4	0.013	0.076	0.079	-0.067	0.057	0.054	0.079	0.061	76.98	21.11	77.57	65.26	0.820	0.201	0.394	0.317	13	19	14	17	17
DLGP3_MOUSE	DLGAP3	Disks large-associated protein 3	0.047	0.102	-0.077	-0.208	0.071	0.065	0.174	0.145	66.90	19.86	69.92	30.51	0.521	0.176	0.656	0.221	1	4	6	5	3
ODO2_MOUSE	DLST	Dihydrolipoyllysine-residue succinyltransferase component of 2-oxoglutarate dehydrogenase complex	0.177	-0.065	-0.162	0.067	0.052	0.085	0.139	0.081	11.25	45.18	48.33	51.06	0.018	0.475	0.322	0.433	5	6	5	4	5
DNJC5_MOUSE	DNAJC5	DnaJ homolog subfamily C member 5	-0.066	-0.276	0.090	-0.748	0.071	0.080	0.090	0.089	62.80	0.00	77.57	0.00	0.384	0.010	0.350	0.000	4	6	4	5	3
DYN1_MOUSE	DNM1	Dynamin-1	-0.124	-0.048	-0.028	0.074	0.056	0.059	0.073	0.082	36.04	46.49	75.06	51.06	0.062	0.443	0.736	0.392	18	26	20	24	17
DPP10_MOUSE	DPP10	Inactive dipeptidyl peptidase 10	-0.023	0.036	-0.148	-0.068	0.089	0.108	0.110	0.083	69.52	40.16	48.33	30.51	0.803	0.747	0.258	0.431	3	3	5	4	5
DPP6_MOUSE	DPP6	Dipeptidyl aminopeptidase-like protein 6	-0.070	0.043	0.149	-0.132	0.048	0.079	0.069	0.098	51.02	36.56	77.57	56.85	0.184	0.606	0.060	0.230	3	9	6	4	5
DPYL2_MOUSE	DPYSL2	Dihydropyrimidinase-related protein 2	-0.084	-0.148	-0.304	0.230	0.068	0.069	0.296	0.200	53.37	9.59	48.33	33.94	0.260	0.082	0.329	0.282	11	9	9	10	9
DYHC1_MOUSE	DYNC1H1	Cytoplasmic dynein 1 heavy chain 1	0.024	-0.068	-0.043	0.198	0.053	0.082	0.077	0.026	73.92	45.18	72.70	11.39	0.662	0.432	0.569	0.000	3	8	7	11	12
EF1A1_MOUSE	EEF1A1	Elongation factor 1-alpha 1	0.026	0.017	0.151	0.089	0.060	0.040	0.073	0.099	73.92	>100.00	77.57	51.06	0.672	0.679	0.072	0.399	3	6	6	5	5
EF1A2_MOUSE	EEF1A2	Elongation factor 1-alpha 2	-0.059	0.023	0.074	-0.023	0.042	0.051	0.074	0.053	53.37	40.16	77.57	>100.00	0.210	0.663	0.414	0.683	4	4	4	4	4
ENOA_MOUSE	ENO1	Alpha-enolase	-0.099	-0.209	-0.276	0.205	0.091	0.070	0.302	0.175	55.36	2.29	48.33	33.94	0.314	0.018	0.400	0.279	10	12	10	11	12
ENOG_MOUSE	ENO2	Gamma-enolase	-0.071	-0.089	-0.186	0.151	0.078	0.048	0.228	0.158	62.80	34.22	48.33	42.52	0.390	0.129	0.443	0.370	7	6	8	9	9
E41L1_MOUSE	EPB41L1	Band 4.1-like protein 1	0.011	0.147	-0.084	0.013	0.094	0.098	0.086	0.131	76.98	19.86	70.88	>100.00	0.910	0.174	0.373	0.926	1	12	4	4	3
E41L2_MOUSE	EPB41L2	Band 4.1-like protein 2	-0.020	0.123	0.178	0.250	0.081	0.045	0.110	0.097	69.52	13.24	77.57	21.42	0.809	0.026	0.189	0.033	2	9	10	7	3
EPHA4_MOUSE	EPHA4	Ephrin type-A receptor 4	0.056	0.020	-0.137	0.069	0.048	0.068	0.054	0.056	50.52	>100.00	48.33	51.06	0.280	0.781	0.049	0.261	4	4	6	7	3
ERC2_MOUSE	ERC2	ERC protein 2	-0.039	0.046	0.075	-0.059	0.049	0.051	0.098	0.076	66.90	33.34	77.57	65.26	0.464	0.402	0.443	0.465	5	14	10	11	7
FUMH_MOUSE	FH	Fumarate hydratase	-0.097	0.004	0.031	0.115	0.074	0.060	0.068	0.084	51.02	>100.00	84.42	42.52	0.226	0.955	0.663	0.212	5	7	2	4	3
FLOT1_MOUSE	FLOT1	Flotillin-1	0.024	0.040	0.155	0.194	0.080	0.090	0.116	0.089	76.88	38.09	77.57	27.41	0.770	0.674	0.288	0.067	2	6	4	5	3
FLOT2_MOUSE	FLOT2	Flotillin-2	0.039	0.257	0.141	-0.008	0.048	0.062	0.071	0.066	59.73	3.50	77.57	>100.00	0.441	0.003	0.089	0.902	3	5	5	5	2
GBRB3_MOUSE	GABRB3	Gamma-aminobutyric acid receptor subunit beta-3	0.113	0.076	0.027	-0.091	0.046	0.064	0.100	0.084	25.67	22.83	84.42	63.03	0.039	0.282	0.786	0.316	2	5	4	3	2
NEUM_MOUSE	GAP43	Neuromodulin	-0.049	0.025	0.069	-0.294	0.077	0.083	0.134	0.098	66.90	>100.00	77.57	3.09	0.549	0.775	0.656	0.034	9	11	12	13	8
G3P_MOUSE	GAPDH	Glyceraldehyde-3-phosphate dehydrogenase	-0.118	0.166	0.242	0.254	0.084	0.089	0.132	0.173	51.02	13.24	77.57	27.41	0.212	0.118	0.094	0.191	14	16	15	13	14
GUAD_MOUSE	GDA	Guanine deaminase	-0.205	0.004	-0.189	0.159	0.089	0.128	0.180	0.182	32.80	>100.00	48.33	42.52	0.067	0.978	0.319	0.416	2	2	3	3	3
GDIA_MOUSE	GDI1	Rab GDP dissociation inhibitor alpha	-0.084	-0.131	-0.179	-0.054	0.096	0.054	0.199	0.162	62.80	9.59	48.33	>100.00	0.407	0.042	0.404	0.746	4	8	6	6	6
CXA1_MOUSE	GJA1	Gap junction alpha-1 protein	0.084	0.083	0.069	0.076	0.079	0.072	0.120	0.095	50.18	22.38	77.57	51.06	0.316	0.280	0.588	0.454	3	8	5	5	4
DHE3_MOUSE	GLUD1	Glutamate dehydrogenase 1	0.020	0.118	-0.100	-0.045	0.045	0.054	0.090	0.078	75.99	15.16	61.72	>100.00	0.683	0.084	0.338	0.580	4	12	7	11	11
GLNA_MOUSE	GLUL	Glutamine synthetase	0.017	0.077	0.103	-0.063	0.053	0.056	0.097	0.086	76.88	21.11	77.57	65.26	0.753	0.239	0.303	0.492	15	16	18	16	14
GNA11_MOUSE	GNA11	Guanine nucleotide-binding protein subunit alpha-11	0.035	0.166	0.111	-0.314	0.088	0.067	0.093	0.079	73.92	8.39	77.57	0.00	0.706	0.044	0.341	0.008	2	5	4	5	3
GNA13_MOUSE	GNA13	Guanine nucleotide-binding protein subunit alpha-13	0.157	-0.085	0.087	-0.041	0.063	0.065	0.128	0.090	25.67	40.16	77.57	>100.00	0.043	0.243	0.525	0.666	3	7	6	7	7
GNAI2_MOUSE	GNAI2	Guanine nucleotide-binding protein G(i), alpha-2 subunit	0.002	-0.002	0.022	-0.027	0.096	0.097	0.097	0.086	>100.00	>100.00	84.42	>100.00	0.983	0.984	0.846	0.768	3	5	6	4	6
GNAO_MOUSE	GNAO1	Guanine nucleotide-binding protein G(o) subunit alpha	0.015	0.052	0.183	-0.248	0.068	0.044	0.104	0.062	76.98	30.09	77.57	3.35	0.828	0.275	0.183	0.005	13	15	16	15	16
GNAQ_MOUSE	GNAQ	Guanine nucleotide-binding protein G(q) subunit alpha	-0.156	0.130	-0.042	0.123	0.063	0.040	0.058	0.061	32.80	8.39	48.33	33.94	0.043	0.021	0.468	0.107	5	7	5	6	6
GNAS1_MOUSE	GNAS1	Guanine nucleotide-binding protein G(s) subunit alpha isoforms XLas	-0.147	0.087	0.024	0.045	0.055	0.052	0.123	0.035	32.80	21.11	84.42	51.06	0.028	0.164	0.857	0.234	5	4	4	7	8
GNAZ_MOUSE	GNAZ	Guanine nucleotide-binding protein G(z) subunit alpha	0.029	0.209	0.053	0.057	0.049	0.068	0.052	0.061	71.60	4.06	78.66	51.06	0.572	0.026	0.322	0.378	5	8	8	7	9
GBB1_MOUSE	GNB1	Guanine nucleotide-binding protein G(I)/G(S)/G(T) subunit beta-1	-0.061	-0.005	0.103	0.198	0.083	0.081	0.130	0.073	66.90	>100.00	77.57	21.15	0.478	0.951	0.463	0.027	3	3	4	5	2
GBG2_MOUSE	GNG2	Guanine nucleotide-binding protein G(I)/G(S)/G(O) subunit gamma-2	-0.009	0.021	0.071	0.322	0.099	0.080	0.073	0.071	73.26	>100.00	77.57	3.09	0.931	0.804	0.397	0.002	3	4	4	4	4
GBG3_MOUSE	GNG3	Guanine nucleotide-binding protein G(I)/G(S)/G(O) subunit gamma-3	0.057	0.130	0.166	0.152	0.056	0.072	0.182	0.086	52.48	19.86	77.57	33.94	0.351	0.108	0.378	0.119	2	3	3	4	5
GBG7_MOUSE	GNG7	Guanine nucleotide-binding protein G(I)/G(S)/G(O) subunit gamma-7	-0.091	0.043	0.120	0.007	0.121	0.044	0.105	0.119	66.90	33.34	77.57	>100.00	0.475	0.365	0.307	0.958	2	3	4	5	3
AATC_MOUSE	GOT1	Aspartate aminotransferase, cytoplasmic	0.010	-0.186	-0.133	0.046	0.083	0.066	0.166	0.136	76.98	5.18	54.74	53.42	0.909	0.025	0.493	0.746	5	3	2	6	6
AATM_MOUSE	GOT2	Aspartate aminotransferase	0.018	0.175	0.137	0.106	0.076	0.062	0.182	0.097	76.98	6.84	77.57	42.52	0.823	0.028	0.445	0.308	14	16	15	18	14
GPDM_MOUSE	GPD2	Glycerol-3-phosphate dehydrogenase	0.075	0.096	-0.152	-0.003	0.067	0.048	0.090	0.095	50.18	19.86	48.33	>100.00	0.305	0.081	0.129	0.974	10	19	20	20	14
G6PI_MOUSE	GPI	Glucose-6-phosphate isomerase	-0.090	-0.051	-0.084	0.140	0.046	0.099	0.229	0.135	42.55	48.71	69.92	42.52	0.091	0.629	0.728	0.344	5	13	9	11	8
GPM6A_MOUSE	GPM6A	Neuronal membrane glycoprotein M6-a	-0.252	0.035	0.200	-0.254	0.069	0.049	0.155	0.113	14.31	36.56	77.57	10.42	0.007	0.512	0.221	0.058	7	9	4	6	7
GPM6B_MOUSE	GPM6B	Neuronal membrane glycoprotein M6-b	-0.106	-0.204	0.133	-0.471	0.055	0.069	0.125	0.092	42.55	4.41	77.57	0.00	0.094	0.019	0.327	0.002	5	8	6	6	5
GRIN1_MOUSE	GPRIN1	G protein-regulated inducer of neurite outgrowth 1	-0.032	0.039	0.114	0.071	0.040	0.036	0.071	0.074	66.90	33.34	77.57	51.06	0.437	0.316	0.133	0.383	9	17	19	18	10
GRIA1_MOUSE	GRIA1	Glutamate receptor 1	-0.021	0.090	0.099	-0.033	0.076	0.070	0.123	0.083	69.52	21.11	77.57	>100.00	0.793	0.247	0.453	0.700	7	12	12	14	12
GRIA2_MOUSE	GRIA2	Glutamate receptor 2	0.015	0.044	0.077	0.048	0.050	0.052	0.072	0.035	76.88	33.34	77.57	51.06	0.766	0.427	0.301	0.213	20	22	19	19	16
GRIA3_MOUSE	GRIA3	Glutamate receptor 3	-0.067	-0.129	0.015	0.075	0.125	0.094	0.077	0.087	66.90	9.19	77.57	51.06	0.608	0.221	0.847	0.414	1	5	3	6	4
NMDZ1_MOUSE	GRIN1	Glutamate [NMDA] receptor subunit zeta-1	0.013	0.041	0.164	0.026	0.089	0.048	0.080	0.073	76.98	35.33	77.57	57.99	0.888	0.424	0.069	0.730	6	12	6	7	5
NMDE2_MOUSE	GRIN2B	Glutamate [NMDA] receptor subunit epsilon-2	-0.010	0.085	0.155	-0.033	0.060	0.037	0.097	0.064	69.45	19.86	77.57	42.52	0.879	0.051	0.137	0.622	7	12	9	8	6
GRM2_MOUSE	GRM2	Metabotropic glutamate receptor 2	0.091	-0.089	0.145	-0.142	0.102	0.067	0.203	0.059	53.37	40.16	77.57	54.22	0.404	0.219	0.559	0.055	2	5	6	4	4

GRM3_MOUSE	GRM3	Metabotropic glutamate receptor 3	-0.109	0.075	0.157	0.165	0.082	0.100	0.144	0.152	51.02	30.67	77.57	36.77	0.219	0.477	0.321	0.312	1	5	4	6	4
GRM5_MOUSE	GRM5	Metabotropic glutamate receptor 5	-0.093	0.075	0.111	-0.146	0.062	0.069	0.068	0.066	51.02	24.72	77.57	30.51	0.175	0.311	0.171	0.087	4	7	8	8	7
GSTM5_MOUSE	GSTM5	Glutathione S-transferase Mu 5	-0.052	-0.096	-0.335	-0.003	0.118	0.103	0.218	0.151	67.56	42.52	48.33	>100.00	0.675	0.390	0.183	0.986	3	3	6	2	3
ECHA_MOUSE	HADHA	Trifunctional enzyme subunit alpha	0.091	-0.019	-0.018	0.023	0.056	0.080	0.146	0.085	35.78	22.38	82.63	>100.00	0.145	0.822	0.910	0.792	3	7	6	3	5
ECHB_MOUSE	HADHB	Trifunctional enzyme subunit beta	0.208	0.170	0.026	-0.006	0.061	0.095	0.081	0.135	13.12	13.24	84.42	>100.00	0.015	0.133	0.760	0.966	3	4	5	3	3
HPLN1_MOUSE	HAPLN1	Hyaluronan and proteoglycan link protein 1	-0.005	0.201	0.163	0.072	0.099	0.071	0.172	0.118	75.25	5.52	77.57	51.06	0.962	0.023	0.352	0.560	6	10	10	11	8
HBA_MOUSE	HBA	Hemoglobin subunit alpha	0.174	0.179	-0.067	0.115	0.043	0.057	0.073	0.102	13.12	5.52	71.59	42.52	0.004	0.018	0.417	0.302	2	3	4	3	3
HXK1_MOUSE	HK1	Hexokinase-1	0.139	0.004	-0.147	0.123	0.067	0.029	0.113	0.051	25.67	>100.00	48.33	33.94	0.075	0.906	0.300	0.043	25	36	31	31	26
NTRI_MOUSE	HNT	Neurotrimin	-0.075	0.196	0.137	-0.018	0.040	0.059	0.122	0.059	51.02	4.06	77.57	>100.00	0.101	0.015	0.311	0.770	5	9	8	6	6
HOME1_MOUSE	HOMER1	Homer protein homolog 1	-0.042	-0.016	0.094	-0.092	0.072	0.076	0.087	0.100	66.90	>100.00	77.57	64.08	0.586	0.837	0.305	0.385	4	9	11	5	5
RASH_MOUSE	HRAS	GTPase HRas	-0.090	-0.086	0.068	0.023	0.100	0.064	0.087	0.071	62.80	40.16	77.57	57.99	0.394	0.229	0.424	0.759	4	3	4	4	3
HS90A_MOUSE	HSP90AA1	Heat shock protein HSP 90-alpha	-0.083	-0.146	-0.084	0.092	0.084	0.073	0.165	0.121	61.66	12.05	71.00	51.06	0.353	0.095	0.615	0.478	8	10	7	12	6
HS90B_MOUSE	HSP90AB1	Heat shock protein HSP 90-beta	0.007	-0.083	0.058	0.025	0.066	0.099	0.146	0.143	76.98	43.44	81.64	>100.00	0.913	0.427	0.702	0.865	3	4	5	4	5
ENPL_MOUSE	HSP90B1	Endoplasmin	-0.021	0.071	0.115	0.181	0.067	0.089	0.080	0.097	69.52	30.53	77.57	27.41	0.765	0.458	0.201	0.108	2	4	5	4	3
GRP78_MOUSE	HSPA5	78 kDa glucose-regulated protein	0.026	0.087	0.002	0.037	0.069	0.073	0.071	0.046	76.35	21.11	>100.00	52.60	0.723	0.277	0.979	0.458	2	10	9	7	7
HSP7C_MOUSE	HSPA8	Heat shock cognate 71 kDa protein	-0.052	-0.069	-0.049	0.114	0.033	0.036	0.082	0.076	51.02	40.16	70.13	37.40	0.159	0.126	0.541	0.193	17	22	18	23	19
GRP75_MOUSE	HSPA9	Stress-70 protein	0.039	0.050	-0.028	0.000	0.072	0.075	0.072	0.123	71.60	35.33	75.06	>100.00	0.605	0.528	0.721	1.000	3	3	4	3	5
CH10_MOUSE	HSPE1	10 kDa heat shock protein	0.122	-0.279	-0.397	0.204	0.110	0.122	0.239	0.150	47.77	4.26	48.33	33.94	0.303	0.053	0.142	0.212	4	4	3	2	3
ICAM5_MOUSE	ICAM5	Intercellular adhesion molecule 5	-0.016	0.173	0.105	-0.118	0.074	0.039	0.101	0.086	69.52	4.06	77.57	56.85	0.832	0.003	0.322	0.211	7	11	11	11	13
IDH3A_MOUSE	IDH3A	Isocitrate dehydrogenase [NAD] subunit alpha	0.165	-0.265	-0.291	-0.053	0.074	0.083	0.241	0.173	25.67	0.00	48.33	>100.00	0.078	0.014	0.297	0.767	8	7	7	8	6
IDH3G_MOUSE	IDH3G	Isocitrate dehydrogenase [NAD] subunit gamma	0.114	0.025	0.125	-0.176	0.086	0.081	0.102	0.171	38.56	>100.00	77.57	56.85	0.241	0.768	0.239	0.343	3	4	3	3	1
IGSF8_MOUSE	IGSF8	Immunoglobulin superfamily member 8	-0.016	0.099	0.154	0.099	0.092	0.066	0.083	0.074	69.52	19.86	77.57	42.52	0.868	0.186	0.091	0.247	5	7	6	6	6
IMMT_MOUSE	IMMT	Mitochondrial inner membrane protein	0.157	0.092	-0.073	-0.077	0.060	0.074	0.067	0.106	25.67	21.11	71.00	65.26	0.035	0.254	0.381	0.488	10	18	14	15	20
AINX_MOUSE	INA	Alpha-internexin	-0.060	-0.007	0.129	0.044	0.097	0.069	0.118	0.065	66.90	>100.00	77.57	51.06	0.554	0.929	0.291	0.516	5	11	10	13	8
IQEC2_MOUSE	IQSEC2	IQ motif and SEC7 domain-containing protein 2	0.068	-0.142	0.080	0.008	0.098	0.083	0.085	0.179	60.32	19.86	77.57	>100.00	0.512	0.128	0.352	0.964	1	4	4	4	2
KCAB2_MOUSE	KCNAB2	Voltage-gated potassium channel subunit beta-2	-0.021	0.239	0.135	0.157	0.058	0.063	0.101	0.069	69.52	3.50	77.57	27.41	0.729	0.007	0.267	0.055	2	6	5	3	5
L1CAM_MOUSE	L1CAM	Neural cell adhesion molecule L1	-0.182	-0.095	0.136	0.022	0.048	0.036	0.123	0.076	14.31	22.83	77.57	>100.00	0.006	0.035	0.370	0.787	7	12	13	11	11
LDHA_MOUSE	LDHA	L-lactate dehydrogenase A chain	-0.130	-0.223	-0.071	-0.019	0.127	0.118	0.299	0.164	55.36	9.19	70.13	>100.00	0.343	0.100	0.821	0.910	3	3	2	4	2
LDHB_MOUSE	LDHB	L-lactate dehydrogenase B chain	-0.116	-0.245	-0.302	-0.062	0.042	0.076	0.203	0.133	32.80	0.00	48.33	>100.00	0.024	0.015	0.161	0.664	6	7	4	5	4
LGI1_MOUSE	LGI1	Leucine-rich glioma-inactivated protein 1	-0.023	0.028	0.009	-0.124	0.025	0.068	0.089	0.081	66.90	40.16	84.30	56.85	0.395	0.694	0.916	0.169	6	7	9	6	5
LIGO1_MOUSE	LINGO1	receptor-interacting protein 1	0.091	0.071	0.046	-0.151	0.065	0.072	0.099	0.079	38.56	26.99	81.76	34.59	0.206	0.348	0.646	0.094	6	8	5	7	6
LPPR4_MOUSE	LPPR4	Lipid phosphate phosphatase-related protein type 4	-0.073	0.015	0.045	0.147	0.086	0.071	0.116	0.093	66.90	>100.00	81.76	33.94	0.423	0.843	0.732	0.154	4	6	4	4	5
LRP1_MOUSE	LRP1	Prolow-density lipoprotein receptor-related protein 1	0.017	0.147	-0.002	0.197	0.061	0.123	0.131	0.063	76.88	19.86	>100.00	21.15	0.786	0.285	0.986	0.015	1	6	12	5	3
LSAMP_MOUSE	LSAMP	Limbic system-associated membrane protein	-0.018	0.113	0.242	0.130	0.062	0.072	0.103	0.086	69.52	19.86	77.57	33.94	0.781	0.160	0.064	0.182	5	10	9	10	8
AOFA_MOUSE	MAOA	Amine oxidase [flavin-containing] A	0.022	0.042	0.273	0.056	0.084	0.088	0.104	0.078	76.88	37.23	77.57	51.06	0.798	0.651	0.073	0.492	1	2	4	3	3
MAP1A_MOUSE	MAP1A	Microtubule-associated protein 1A	0.002	0.056	0.029	-0.014	0.023	0.059	0.051	0.039	>100.00	30.67	84.42	>100.00	0.950	0.370	0.583	0.732	7	24	22	22	17
MAP1B_MOUSE	MAP1B	Microtubule-associated protein 1B	-0.151	0.028	-0.054	0.078	0.040	0.049	0.062	0.041	14.31	38.09	70.39	42.52	0.012	0.587	0.374	0.110	5	13	15	20	11
MAP2_MOUSE	MAP2	Microtubule-associated protein 2	-0.048	0.097	-0.077	-0.009	0.097	0.089	0.068	0.074	66.90	21.11	70.88	>100.00	0.634	0.314	0.335	0.909	3	4	2	4	2
MAP6_MOUSE	MAP6	Microtubule-associated protein 6	0.008	0.029	0.043	0.051	0.039	0.040	0.029	0.047	76.98	37.23	81.64	51.06	0.848	0.481	0.168	0.309	10	24	11	21	16
MK01_MOUSE	MAPK1	Mitogen-activated protein kinase 1	0.166	0.143	-0.077	0.219	0.115	0.066	0.109	0.049	35.78	13.24	71.00	16.41	0.191	0.063	0.504	0.002	4	5	3	5	4
MDHC_MOUSE	MDH1	Malate dehydrogenase, cytoplasmic	-0.077	-0.246	-0.190	0.203	0.105	0.076	0.255	0.167	66.90	0.00	48.33	33.94	0.490	0.012	0.475	0.262	7	6	5	5	3
MDHM_MOUSE	MDH2	Malate dehydrogenase	0.020	-0.016	-0.021	0.041	0.034	0.055	0.080	0.031	74.06	>100.00	80.37	51.06	0.578	0.785	0.808	0.226	16	18	13	15	14
MINK1_MOUSE	MINK1	Misshapen-like kinase 1	-0.202	0.194	-0.145	0.009	0.063	0.133	0.181	0.190	13.12	15.16	54.74	>100.00	0.016	0.183	0.426	0.965	1	3	5	4	1
MPP2_MOUSE	MPP2	MAGUK p55 subfamily member 2	-0.098	0.170	-0.136	-0.012	0.162	0.112	0.220	0.131	66.90	15.03	61.72	>100.00	0.567	0.195	0.566	0.928	1	7	5	7	3
MTCH2_MOUSE	MTCH2	Mitochondrial carrier homolog 2	0.076	0.149	0.068	0.056	0.067	0.110	0.149	0.091	50.18	19.86	77.57	51.06	0.291	0.219	0.682	0.560	2	4	4	2	4
MYH10_MOUSE	MYH10	Myosin-10	-0.023	0.038	-0.120	-0.078	0.106	0.084	0.130	0.114	69.52	38.09	58.44	65.26	0.835	0.664	0.378	0.517	2	3	3	3	1
MRLC2_MOUSE	MYL12B	Myosin regulatory light chain MRLC2	-0.014	-0.010	0.055	-0.005	0.065	0.103	0.100	0.080	69.52	>100.00	81.64	>100.00	0.834	0.922	0.608	0.952	2	3	3	3	3
MYO5A_MOUSE	MYO5A	Myosin-Va	-0.055	-0.059	-0.029	0.070	0.109	0.076	0.093	0.087	66.90	45.18	75.06	51.06	0.627	0.462	0.768	0.455	1	7	7	10	7
SNAB_MOUSE	NAPB	Beta-soluble NSF attachment protein	-0.211	-0.208	-0.215	-0.090	0.138	0.111	0.178	0.138	48.32	9.19	48.33	65.26	0.166	0.129	0.274	0.536	2	5	5	7	5
SNAG_MOUSE	NAPG	Gamma-soluble NSF attachment protein	-0.003	-0.204	-0.142	-0.045	0.113	0.085	0.172	0.196	>100.00	4.67	54.74	>100.00	0.979	0.062	0.406	0.826	2	5	4	5	5
NCAM1_MOUSE	NCAM1	Neural cell adhesion molecule 1	-0.175	0.224	0.011	0.007	0.081	0.099	0.156	0.082	36.04	6.84	84.42	>100.00	0.079	0.055	0.948	0.931	3	6	7	5	4
NCAM2_MOUSE	NCAM2	Neural cell adhesion molecule 2	0.115	0.120	0.066	0.115	0.075	0.056	0.101	0.056	35.78	15.16	77.57	33.94	0.165	0.073	0.539	0.074	3	6	6	7	9
NCAN_MOUSE	NCAN	Neurocan core protein	0.033	0.263	0.274	0.067	0.106	0.075	0.198	0.119	76.88	3.50	77.57	51.06	0.769	0.009	0.192	0.593	6	6	7	7	6
NCDN_MOUSE	NCDN	Neurochondrin	0.018	-0.146	-0.130	0.153	0.107	0.100	0.237	0.103	76.98	26.36	61.72	33.94	0.874	0.191	0.618	0.177	1	2	3	4	4
NCKP1_MOUSE	NCKAP1	Nck-associated protein 1	0.071	-0.023	-0.012	0.161	0.097	0.042	0.084	0.071	59.24	>100.00	85.06	27.41	0.489	0.605	0.901	0.064	4	6	7	5	6
NDUAA_MOUSE	NDUFA10	NADH dehydrogenase [ubiquinone] 1 alpha subcomplex subunit 10	0.214	0.162	-0.276	-0.236	0.121	0.106	0.166	0.094	25.67	19.86	48.33	11.02	0.128	0.190	0.208	0.051	2	5	7	4	3
NDUAC_MOUSE	NDUFA12	NADH dehydrogenase [ubiquinone] 1 alpha subcomplex subunit 12	0.083	0.012	-0.094	0.075	0.056	0.091	0.170	0.169	38.56	>100.00	70.88	51.06	0.192	0.896	0.626	0.676	3	6	7	5	4
NDUA4_MOUSE	NDUFA4	NADH dehydrogenase [ubiquinone] 1 alpha subcomplex subunit 4	0.197	-0.011	-0.243	0.002	0.146	0.100	0.166	0.163	35.78	>100.00	48.33	>100.00	0.215	0.915	0.228	0.991	5	6	5	5	5
NDUA5_MOUSE	NDUFA5	NADH dehydrogenase [ubiquinone] 1 alpha subcomplex subunit 5	0.098	0.025	-0.206	0.000	0.093	0.120	0.131	0.138	50.18	>100.00	48.33	>100.00	0.326	0.844	0.192	0.998	3	3	3	2	2
NDUA9_MOUSE	NDUFA9	NADH dehydrogenase [ubiquinone] 1 alpha subcomplex subunit 9	0.203	-0.025	-0.168	-0.103	0.101	0.102	0.159	0.176	25.67	>100.00	48.33	65.26	0.096	0.817	0.350	0.579	3	10	5	8	5
NDUBA_MOUSE	NDUFB10	NADH dehydrogenase [ubiquinone] 1 beta subcomplex subunit 10	0.171	-0.088	-0.150	0.217	0.124	0.100	0.060	0.118	35.78	42.52	48.33	27.41	0.210	0.410	0.041	0.107	2	4	5	5	2
NDUB5_MOUSE	NDUFB5	NADH dehydrogenase [ubiquinone] 1 beta subcomplex subunit 5	0.293	0.084	-0.159	0.059	0.091	0.060	0.184	0.119	13.12	21.11	54.74	51.06	0.014	0.234	0.432	0.634	2	3	4	3	4
NDUB7_MOUSE	NDUFB7	NADH dehydrogenase [ubiquinone] 1 beta subcomplex subunit 7	0.148	0.196	-0.216	0.089	0.112	0.107	0.161	0.154	38.56	13.60	48.33	51.06	0.237	0.134	0.213	0.586	1	4	2	3	4
NDUB8_MOUSE	NDUFB8	NADH dehydrogenase [ubiquinone] 1 beta subcomplex subunit 8	-0.024	-0.022	-0.307	0.188	0.124	0.139	0.118	0.082	69.52	>100.00	48.33	>100.00	0.852	0.879	0.043	0.052	3	3	3	4	3
NDUB9_MOUSE	NDUFB9	NADH dehydrogenase [ubiquinone] 1 beta subcomplex subunit 9	0.012	0.074	-0.039	-0.018	0.093	0.148	0.180	0.132	76.98	35.33	75.29	>100.00	0.897	0.631	0.824	0.899	1	2	4	4	3
NDUC2_MOUSE	NDUFC2	NADH dehydrogenase [ubiquinone] 1 subunit C2	0.122	-0.146	-0.178	-0.511	0.111	0.120	0.165	0.185	47.77	34.22	48.33	0.00	0.305	0.258	0.391	0.027	2	6	4	4	4
NDUS1_MOUSE	NDUFS1	NADH-ubiquinone oxidoreductase 75 kDa subunit	0.130	0.042	-0.163	-0.120	0.086	0.038	0.116	0.090	35.78	33.34	48.33	56.85	0.167	0.312	0.259	0.231	11	20	19	16	12
NDUS2_MOUSE	NDUFS2	NADH dehydrogenase [ubiquinone] iron-sulfur protein 2	0.087	-0.062	-0.073	0.033	0.091	0.079	0.112	0.112	51.06	45.18	71.59	56.93	0.369	0.463	0.590	0.774	6	8	7	7	6
NDUS3_MOUSE	NDUFS3	NADH dehydrogenase [ubiquinone] iron-sulfur protein 3	0.198	0.035	-0.040	0.030	0.105	0.037	0.133	0.135	25.67	35.33	74.92	57.99	0.096	0.385	0.779	0.830	2	7	6	5	4
NDUS6_MOUSE	NDUFS6	NADH dehydrogenase [ubiquinone] iron-sulfur protein 6	0.131	-0.011	-0.095	-0.545	0.168	0.166	0.195	0.179	54.63	>100.00	71.00	0.00	0.472	0.949	0.677	0.017	1	3	3	3	2
NDUS7_MOUSE	NDUFS7	NADH dehydrogenase [ubiquinone] iron-sulfur protein 7	0.121	-0.044	-0.046	-0.184	0.112	0.128	0.161	0.102	47.63	>100.00	73.06	27.41	0.313	0.740	0.793	0.110	1	4	3	4	3
NDUV1_MOUSE	NDUFV1	NADH dehydrogenase [ubiquinone] flavoprotein 1	0.099	0.069	-0.187	0.021	0.084	0.036	0.115	0.064	25.67	21.11	48.33	>100.00	0.292	0.093	0.154	0.758	7	12	11	10	10
NFL_MOUSE	NEFL	Neurofilament light polypeptide	-0.181	0.144	0.108	-0.016	0.152	0.112	0.131	0.080	51.02	19.86	77.57	>100.00	0.279	0.235	0.476	0.850	2	4	3	9	5
NFM_MOUSE	NEFM	Neurofilament medium polypeptide	-0.014	0.035	-0.114	0.164	0.088	0.069	0.158	0.075	69.52	38.09	62.19	27.41	0.878	0.632	0.490	0.071	2	4	6	12	4
NEGR1_MOUSE	NEGR1	Neuronal growth regulator 1	-0.010	0.108	-0.040	0.108	0.061	0.060	0.092	0.046	69.45	19.86	73.69	33.94	0.881	0.111	0.670	0.047	2	3	3	4	2
NFASC_MOUSE	NFASC	Neurofascin	-0.022	0.052	0.145	-0.039	0.045	0.045	0.043	0.047	68.31	30.53	77.57	>100.00	0.636	0.290	0.037	0.439	14	19	17	22	14
NIPS1_MOUSE	NIPSNAP1	Protein NipSnap homolog 1	0.022	0.005	0.128	0.023	0.044	0.071	0.101	0.074	47.77	>100.00	77.57	57.99	0.635	0.940	0.255	0.766	3	3	4	6	6
NLGN2_MOUSE	NLGN2	Neuroligin-2	0.183	0.149	-0.014	-0.076	0.114	0.098	0.114	0.105	31.49	19.86	84.93	65.26	0.150	0.176	0.904	0.491	1	2	6	4	3
NLGN3_MOUSE	NLGN3	Neuroligin-3	0.080	0.137	-0.134	0.084	0.056	0.084	0.101	0.051	38.56	19.86	54.74	42.52	0.196	0.144	0.229	0.140	2	5	5	5	3
NPTN_MOUSE	NPTN	Neuroplastin	0.045	0.096	0.175	0.132	0.078	0.061	0.135	0.089	71.43	19.86	77.57	33.94	0.587	0.154	0.293	0.176	4	7	6	6	6
NPTX1_MOUSE	NPTX1	Neuronal pentraxin-1	0.031	0.169	0.143	0.040	0.070	0.046	0.136	0.094	66.90	4.86	77.57	53.32	0.675	0.007	0.336	0.682	3	6	4	4	5
NRCAM_MOUSE	NRCAM	Neuronal cell adhesion molecule	0.083	0.057	0.044	0.021	0.132	0.077	0.112	0.125	62.13	33.34	81.76	>100.00	0.549	0.491	0.703	0.873	3	6	7	6	5
NSF_MOUSE	NSF	Vesicle-fusing ATPase	-0.106	-0.084	-0.044	0.062	0.050	0.062	0.178	0.131	41.68	40.16	74.92	33.94	0.066	0.246	0.811	0.651	28	32	29	34	31
NOE1_MOUSE	OLFM1	Noelin	-0.089	-0.027	0.116	-0.055	0.042	0.081	0.104	0.092	42.55	>100.00	77.57	>100.00	0.071	0.743	0.324	0.566	2	7	3	6	4
OMGP_MOUSE	OMG	Oligodendrocyte-myelin glycoprotein	-0.085	-0.132	-0.070	-0.284	0.053	0.048	0.108	0.110	51.02	9.19	69.92	5.32	0.146	0.029	0.526	0.044	4	3	4	4	4
OPA1_MOUSE	OPA1	Dynamin-like 120 kDa protein	0.008	0.122	-0.095	-0.086	0.105	0.124	0.178	0.062	>100.00	21.11	70.88	61.67	0.938	0.371	0.584	0.204	2	9	8	10	9

PACN1_MOUSE	PACSIN1	Protein kinase C and casein kinase substrate in neurons protein 1	-0.099	-0.187	-0.149	0.337	0.097	0.085	0.245	0.202	58.53	8.39	55.07	21.15	0.339	0.072	0.561	0.149	6	8	6	7	4
PALM_MOUSE	PALM	Paralemmin	0.023	0.027	0.174	0.074	0.092	0.053	0.074	0.094	76.88	38.86	77.57	51.06	0.812	0.626	0.100	0.453	6	11	10	10	9
148678151	PCDH1	protocadherin 1	0.074	0.023	0.167	-0.023	0.076	0.058	0.073	0.092	52.48	>100.00	77.57	>100.00	0.366	0.707	0.049	0.808	4	8	8	4	9
PCLO_MOUSE	PCLO	Protein piccolo	0.099	0.000	0.163	-0.015	0.085	0.033	0.121	0.047	47.77	>100.00	77.57	>100.00	0.276	0.991	0.252	0.760	5	26	21	25	18
PCSK1_MOUSE	PCSK1N	ProSAAS	-0.232	0.121	-0.073	-0.029	0.190	0.119	0.100	0.153	51.02	21.11	71.00	>100.00	0.282	0.342	0.470	0.853	1	2	3	4	3
ODPA_MOUSE	PDHA1	Pyruvate dehydrogenase E1 component subunit alpha, somatic form	0.229	-0.054	-0.145	-0.115	0.055	0.078	0.105	0.081	13.12	46.49	48.33	56.85	0.010	0.512	0.257	0.194	6	10	10	8	9
ODPB_MOUSE	PDHB	Pyruvate dehydrogenase E1 component subunit beta	0.093	-0.037	-0.146	0.074	0.072	0.073	0.109	0.073	39.76	>100.00	48.33	51.06	0.240	0.623	0.263	0.351	6	9	7	9	8
PDIA3_MOUSE	PDIA3	Protein disulfide-isomerase A3	0.099	0.090	-0.006	0.086	0.036	0.054	0.098	0.039	25.67	19.86	>100.00	42.52	0.024	0.135	0.957	0.067	6	8	9	8	6
K6PF_MOUSE	PFKM	6-phosphofructokinase, muscle type	0.018	0.000	-0.010	-0.068	0.035	0.064	0.049	0.101	73.92	>100.00	85.80	65.26	0.626	1.000	0.861	0.523	5	4	6	3	4
PROF2_MOUSE	PFN2	Profilin-2	0.022	-0.121	-0.384	0.090	0.081	0.169	0.389	0.176	76.88	43.44	48.33	51.06	0.795	0.500	0.329	0.625	2	3	3	3	1
PGAM1_MOUSE	PGAM1	Phosphoglycerate mutase 1	0.001	-0.062	-0.176	0.059	0.075	0.077	0.263	0.167	>100.00	45.18	54.74	52.60	0.993	0.466	0.543	0.734	4	10	6	10	5
PGRC1_MOUSE	PGRMC1	Membrane-associated progesterone receptor component 1	-0.172	0.043	-0.156	-0.074	0.120	0.098	0.067	0.122	51.02	38.09	48.33	65.26	0.195	0.680	0.046	0.565	2	4	5	4	2
PHB_MOUSE	PHB	Prohibitin	0.181	0.136	-0.033	0.049	0.071	0.043	0.087	0.073	25.67	8.39	75.29	51.06	0.038	0.014	0.723	0.521	4	8	7	7	5
PHB2_MOUSE	PHB2	Prohibitin-2	0.190	0.085	-0.002	0.048	0.082	0.051	0.126	0.073	25.67	21.11	>100.00	51.06	0.059	0.137	0.989	0.532	3	9	7	7	6
KPYM_MOUSE	PKM2	Pyruvate kinase isozymes M1/M2	-0.016	-0.188	0.194	-0.237	0.103	0.051	0.197	0.077	69.52	2.48	77.57	7.46	0.881	0.012	0.426	0.016	5	5	5	5	5
PKP4_MOUSE	PKP4	Plakophilin-4	0.013	0.062	0.197	0.108	0.074	0.106	0.132	0.099	76.98	35.33	77.57	42.52	0.867	0.586	0.159	0.311	1	5	4	3	3
PLEC1_MOUSE	PLEC1	Plectin-1	0.014	0.098	0.004	0.069	0.086	0.064	0.055	0.073	76.98	19.86	85.31	51.06	0.876	0.164	0.944	0.372	3	13	12	14	6
MYPR_MOUSE	PLP1	Myelin proteolipid protein	-0.172	0.015	0.061	0.277	0.054	0.159	0.193	0.171	12.11	>100.00	81.64	27.41	0.016	0.928	0.743	0.144	7	6	7	6	5
PLXA4_MOUSE	PLXNA4	Plexin-A4	-0.029	0.068	-0.198	-0.063	0.101	0.062	0.180	0.167	69.52	26.36	48.33	>100.00	0.783	0.326	0.387	0.719	1	4	7	4	4
PPIA_MOUSE	PPIA	Peptidyl-prolyl cis-trans isomerase A	-0.130	-0.288	-0.253	0.050	0.061	0.072	0.265	0.211	38.56	0.00	48.33	56.85	0.070	0.004	0.370	0.817	6	9	7	8	4
2AAA_MOUSE	PPP2R1A	alpha isoform	-0.131	-0.130	-0.043	0.196	0.078	0.107	0.066	0.153	48.32	35.97	72.70	33.94	0.139	0.258	0.551	0.239	5	5	3	3	5
PP2BA_MOUSE	PPP3CA	Serine/threonine-protein phosphatase 2B catalytic subunit alpha isoform	0.013	-0.093	-0.189	0.099	0.131	0.094	0.191	0.170	76.98	42.18	48.33	51.06	0.922	0.367	0.352	0.575	4	5	4	3	3
CANB1_MOUSE	PPP3R1	Calcineurin subunit B type 1	-0.053	-0.011	-0.129	0.218	0.106	0.065	0.232	0.186	66.90	>100.00	62.19	33.94	0.637	0.864	0.616	0.276	3	4	4	3	4
PRDX1_MOUSE	PRDX1	Peroxiredoxin-1	0.062	0.090	0.145	0.335	0.054	0.072	0.071	0.053	50.10	21.11	77.57	0.00	0.286	0.256	0.075	0.002	6	7	5	5	6
PRDX5_MOUSE	Prdx5	Peroxiredoxin-5	0.056	-0.593	-0.382	-0.399	0.070	0.044	0.149	0.139	58.94	0.00	48.33	0.00	0.448	9.096	0.044	0.021	4	6	6	4	4
PRDX6_MOUSE	PRDX6	Peroxiredoxin-6	-0.013	-0.027	0.012	0.254	0.077	0.098	0.123	0.097	69.52	>100.00	84.42	21.42	0.871	0.790	0.925	0.038	4	6	5	4	4
KAPCA_MOUSE	PRKACA	cAMP-dependent protein kinase catalytic subunit alpha	-0.109	-0.096	0.041	0.177	0.099	0.081	0.164	0.109	53.96	40.16	84.42	33.94	0.316	0.273	0.818	0.146	1	3	3	3	1
KAP3_MOUSE	PRKAR2B	cAMP-dependent protein kinase type II-beta regulatory subunit	-0.045	-0.027	0.075	0.049	0.117	0.130	0.114	0.115	68.31	>100.00	77.57	52.60	0.715	0.841	0.516	0.690	1	3	4	3	1
KPCB_MOUSE	PRKCB1	Protein kinase C beta type	0.045	0.008	0.064	0.203	0.051	0.035	0.098	0.092	58.53	>100.00	77.57	27.41	0.412	0.815	0.577	0.058	3	5	3	5	4
KPCG_MOUSE	PRKCG	Protein kinase C gamma type	-0.044	-0.099	0.023	-0.080	0.031	0.068	0.094	0.065	55.36	35.97	84.42	63.47	0.198	0.181	0.831	0.250	9	16	13	16	13
PRIO_MOUSE	PRNP	Major prion protein	-0.060	-0.015	0.014	-0.217	0.061	0.111	0.113	0.129	62.80	>100.00	84.42	22.68	0.355	0.896	0.908	0.144	5	5	5	5	3
E9PUL5_MOUSE	PRRT2	proline-rich transmembrane protein 2	-0.063	0.380	0.153	-0.238	0.081	0.077	0.091	0.116	66.90	0.00	77.57	13.71	0.459	0.002	0.164	0.106	3	3	2	3	1
PYGB_MOUSE	PYGB	Glycogen phosphorylase, brain form	0.100	-0.034	-0.118	0.003	0.122	0.070	0.108	0.149	54.63	>100.00	54.74	>100.00	0.437	0.647	0.346	0.985	4	5	9	8	10
RAB10_MOUSE	RAB10	Ras-related protein Rab-10	-0.017	0.148	-0.072	0.156	0.075	0.116	0.063	0.068	69.52	19.86	71.00	27.41	0.833	0.240	0.290	0.070	4	3	5	5	2
RAB14_MOUSE	RAB14	Ras-related protein Rab-14	-0.035	0.082	0.003	0.127	0.043	0.059	0.109	0.051	66.90	13.24	85.56	33.94	0.441	0.202	0.982	0.038	5	5	4	3	3
RAB1A_MOUSE	RAB1A	Ras-related protein Rab-1A	-0.103	0.034	0.002	0.191	0.088	0.108	0.084	0.068	53.37	40.16	85.56	21.15	0.301	0.760	0.981	0.023	2	4	4	5	3
RAB1B_MOUSE	RAB1B	Ras-related protein Rab-1B	-0.162	-0.027	-0.064	0.061	0.055	0.074	0.131	0.116	27.55	>100.00	70.39	51.06	0.021	0.727	0.612	0.613	1	3	3	3	3
RAB2A_MOUSE	RAB2A	Ras-related protein Rab-2A	0.020	-0.102	0.016	0.036	0.039	0.041	0.071	0.053	73.92	19.86	84.42	52.60	0.622	0.040	0.837	0.519	5	7	7	7	6
RAB35_MOUSE	RAB35	Ras-related protein Rab-35	-0.023	-0.051	0.141	-0.201	0.049	0.118	0.234	0.138	68.31	>100.00	77.57	30.51	0.649	0.684	0.615	0.189	2	4	3	3	3
RAB3A_MOUSE	RAB3A	Ras-related protein Rab-3A	-0.050	-0.103	0.025	0.029	0.046	0.021	0.069	0.054	62.80	9.19	84.42	54.22	0.322	0.003	0.750	0.605	6	6	6	6	6
RAB3C_MOUSE	RAB3C	Ras-related protein Rab-3C	-0.074	-0.149	-0.009	-0.174	0.064	0.083	0.072	0.048	55.36	14.05	85.93	11.02	0.288	0.119	0.906	0.015	4	4	5	6	5
RAB7A_MOUSE	RAB7A	Ras-related protein Rab-7a	0.015	0.067	0.115	0.066	0.017	0.030	0.048	0.067	72.00	21.11	77.57	51.06	0.428	0.060	0.069	0.357	5	6	8	9	5
RAC1_MOUSE	RAC1	Ras-related C3 botulinum toxin substrate 1	0.078	-0.329	0.244	-0.594	0.104	0.134	0.184	0.149	58.94	2.48	77.57	0.00	0.474	0.040	0.271	0.011	2	4	3	3	4
RALA_MOUSE	RALA	Ras-related protein Ral-A	-0.078	0.088	-0.087	0.235	0.080	0.077	0.113	0.100	51.02	22.38	70.88	21.15	0.360	0.307	0.443	0.064	3	3	1	4	4
RAP2B_MOUSE	RAP2B	Ras-related protein Rap-2b	0.053	-0.009	0.064	0.178	0.061	0.078	0.124	0.058	56.97	>100.00	78.34	22.04	0.408	0.915	0.653	0.023	3	4	4	3	3
RHOA_MOUSE	RHOA	Transforming protein RhoA	-0.002	-0.112	-0.064	0.342	0.094	0.163	0.142	0.185	>100.00	43.91	70.39	21.15	0.981	0.523	0.660	0.128	3	5	3	4	4
RHOB_MOUSE	RHOB	Rho-related GTP-binding protein RhoB	0.021	0.123	0.089	-0.077	0.072	0.089	0.110	0.113	76.88	19.86	77.57	65.26	0.779	0.213	0.484	0.521	4	4	3	2	3
RP3A_MOUSE	RPH3A	Rabphilin-3A	-0.019	-0.263	0.011	-0.021	0.039	0.096	0.100	0.081	68.31	2.48	84.42	>100.00	0.633	0.027	0.921	0.800	7	8	6	7	6
RTN1_MOUSE	RTN1	Reticulon-1	-0.034	-0.068	-0.053	-0.048	0.076	0.084	0.082	0.079	68.31	45.18	70.02	>100.00	0.668	0.448	0.507	0.560	4	5	5	6	4
SAM50_MOUSE	SAMM50	Sorting and assembly machinery component 50 homolog	0.192	0.195	-0.133	-0.014	0.108	0.070	0.140	0.081	25.67	5.42	54.74	>100.00	0.118	0.031	0.347	0.863	1	4	3	2	3
CI126_MOUSE	SCAI	UPF0682 protein C9orf126 homolog	0.106	0.230	0.054	0.161	0.062	0.056	0.136	0.070	32.80	>100.00	81.64	27.41	0.145	0.004	0.709	0.069	2	5	5	5	4
SCAM1_MOUSE	SCAMP1	Secretory carrier-associated membrane protein 1	-0.076	0.022	-0.199	-0.261	0.148	0.103	0.191	0.085	66.90	>100.00	48.33	5.51	0.622	0.840	0.312	0.016	2	3	3	2	3
B1AWN6_MOUSE	SCN2A1	Sodium channel, voltage-gated, type II, alpha 1	0.003	0.127	0.015	0.003	0.086	0.082	0.078	0.067	>100.00	19.86	84.42	>100.00	0.976	0.161	0.843	0.962	2	5	6	7	2
DHSA_MOUSE	SDHA	Succinate dehydrogenase [ubiquinone] flavoprotein subunit	0.142	-0.098	-0.117	-0.353	0.076	0.111	0.104	0.112	25.67	42.52	54.74	0.00	0.111	0.420	0.297	0.016	4	11	8	7	6
DHSB_MOUSE	SDHB	Succinate dehydrogenase [ubiquinone] iron-sulfur subunit	0.155	0.187	-0.051	0.165	0.096	0.082	0.135	0.129	33.15	8.39	70.52	33.94	0.152	0.058	0.746	0.240	4	9	7	7	5
SEPT3_MOUSE	SEPT3	Neuronal-specific septin-3	0.095	-0.126	0.183	0.042	0.034	0.030	0.088	0.091	25.67	5.32	77.57	52.60	0.025	0.003	0.075	0.656	6	8	6	4	6
SEPT5_MOUSE	SEPT5	Septin-5	0.005	-0.005	0.075	-0.098	0.068	0.056	0.040	0.070	>100.00	>100.00	77.57	60.33	0.944	0.934	0.094	0.227	8	10	7	9	7
SEPT7_MOUSE	SEPT7	Septin-7	-0.026	0.017	0.066	0.000	0.064	0.034	0.047	0.037	68.53	>100.00	77.57	>100.00	0.696	0.636	0.187	0.994	10	12	10	13	12
SEPT8_MOUSE	SEPT8	Septin-8	-0.021	0.092	0.159	0.099	0.056	0.024	0.110	0.080	69.52	13.62	77.57	42.52	0.724	0.010	0.268	0.254	4	8	7	7	7
SFXN1_MOUSE	SFXN1	Sideroflexin-1	0.069	0.287	0.041	-0.049	0.102	0.091	0.128	0.095	60.32	3.50	82.66	>100.00	0.516	0.017	0.763	0.623	2	5	3	4	2
SFXN3_MOUSE	SFXN3	Sideroflexin-3	0.231	0.048	0.006	0.116	0.087	0.081	0.134	0.142	20.99	35.33	85.19	51.06	0.037	0.579	0.967	0.449	7	10	10	9	7
SFXN5_MOUSE	SFXN5	Sideroflexin-5	0.001	0.052	-0.096	0.008	0.066	0.109	0.146	0.151	>100.00	36.56	70.10	>100.00	0.986	0.652	0.556	0.956	4	9	7	7	5
SH3G2_MOUSE	SH3GL2	Endophilin-A1	-0.101	-0.070	-0.228	0.123	0.042	0.104	0.265	0.208	36.04	45.42	48.33	51.06	0.044	0.529	0.458	0.570	4	10	8	10	5
S12A5_MOUSE	SLC12A5	Solute carrier family 12 member 5	-0.096	-0.035	0.073	-0.176	0.044	0.034	0.071	0.074	41.68	47.31	77.57	18.51	0.063	0.352	0.327	0.056	17	29	25	27	19
VGLU1_MOUSE	SLC17A7	Vesicular glutamate transporter 1	-0.181	-0.124	0.070	-0.186	0.042	0.079	0.121	0.109	32.31	27.47	77.57	30.51	0.004	0.164	0.558	0.126	4	5	3	6	6
EAA2_MOUSE	SLC1A2	Excitatory amino acid transporter 2	-0.074	0.090	0.115	-0.102	0.077	0.050	0.118	0.070	62.80	19.86	77.57	59.17	0.373	0.109	0.347	0.184	10	13	10	12	8
EAA1_MOUSE	SLC1A3	Excitatory amino acid transporter 1	-0.116	0.240	-0.025	-0.166	0.077	0.084	0.161	0.169	51.02	4.06	80.37	56.85	0.171	0.023	0.894	0.354	5	5	6	6	7
M2OM_MOUSE	SLC25A11	Mitochondrial 2-oxoglutarate/malate carrier protein	0.179	0.130	-0.170	0.028	0.080	0.088	0.107	0.121	25.67	19.86	48.33	57.99	0.058	0.193	0.143	0.825	7	9	9	5	9
CMC1_MOUSE	SLC25A12	Calcium-binding mitochondrial carrier protein Aralar1	0.090	0.115	-0.119	0.052	0.076	0.082	0.140	0.113	47.77	19.86	58.44	52.60	0.284	0.198	0.449	0.660	11	16	11	9	12
GHC1_MOUSE	SLC25A22	Mitochondrial glutamate carrier 1	0.134	-0.111	-0.225	-0.202	0.104	0.056	0.153	0.197	38.56	24.02	48.33	56.85	0.232	0.083	0.190	0.336	4	5	5	6	5
MPCP_MOUSE	SLC25A3	Phosphate carrier protein	0.208	0.072	-0.172	0.108	0.101	0.111	0.095	0.173	25.67	33.34	48.33	51.06	0.086	0.550	0.166	0.553	6	10	11	11	11
ADT1_MOUSE	SLC25A4	ADP/ATP translocase 1	0.140	0.050	-0.245	0.102	0.114	0.103	0.156	0.166	38.56	36.56	48.33	51.06	0.255	0.648	0.234	0.561	7	10	7	7	7
ADT2_MOUSE	SLC25A5	ADP/ATP translocase 2	0.174	0.113	-0.118	0.228	0.125	0.094	0.088	0.122	35.78	21.11	54.74	27.41	0.202	0.270	0.245	0.111	7	10	6	8	6
GTR1_MOUSE	SLC2A1	Solute carrier family 2, facilitated glucose transporter member 1	-0.152	0.094	0.194	-0.227	0.072	0.060	0.148	0.071	36.04	19.86	77.57	7.23	0.068	0.154	0.301	0.013	3	3	3	4	2
GTR3_MOUSE	SLC2A3	Solute carrier family 2, facilitated glucose transporter member 3	0.074	0.121	0.143	-0.115	0.064	0.084	0.067	0.031	50.18	19.86	77.57	30.51	0.294	0.188	0.108	0.007	4	5	6	7	5
4F2_MOUSE	SLC3A2	4F2 cell-surface antigen heavy chain	-0.033	-0.019	0.069	-0.113	0.056	0.061	0.107	0.064	66.90	>100.00	77.57	56.85	0.566	0.768	0.550	0.133	11	14	14	14	14
S4A4_MOUSE	SLC4A4	Electrogenic sodium bicarbonate cotransporter 1	-0.035	0.150	0.184	-0.056	0.048	0.051	0.124	0.056	66.90	7.33	77.57	65.26	0.489	0.030	0.226	0.358	3	6	10	7	7
SC6A1_MOUSE	SLC6A1	Sodium- and chloride-dependent GABA transporter 1	-0.011	-0.033	0.039	-0.536	0.058	0.041	0.138	0.138	69.52	22.38	84.42	0.00	0.858	0.449	0.799	0.006	5	5	5	4	5
S6A11_MOUSE	SLC6A11	Sodium- and chloride-dependent GABA transporter 3	-0.113	0.102	-0.052	0.119	0.057	0.067	0.113	0.099	42.55	19.86	70.13	42.52	0.086	0.171	0.653	0.265	4	5	6	7	6
NAC1_MOUSE	SLC8A1	Sodium/calcium exchanger 1	0.019	0.137	0.210	0.132	0.028	0.037	0.075	0.090	73.52	7.21	77.57	33.94	0.536	0.006	0.022	0.201	6	8	7	7	8
SNP25_MOUSE	SNAP25	Synaptosomal-associated protein 25	0.057	0.169	0.218	0.095	0.051	0.042	0.098	0.077	50.44	4.06	77.57	42.52	0.308	0.005	0.080	0.270	9	14	13	13	11
AP180_MOUSE	SNAP91	Clathrin coat assembly protein AP180	0.025	-0.077	-0.157	0.053	0.099	0.109	0.172	0.158	76.88	45.18	54.74	53.21	0.807	0.509	0.391	0.747	6	9	5	6	5
SYUB_MOUSE	SNCB	Beta-synuclein	-0.188	-0.271	-0.266	0.239	0.080	0.084	0.206	0.157	42.55	0.00	48.33	27.41	0.047	0.012	0.249	0.167	3	4	3	3	3
SODM_MOUSE	SOD2	Superoxide dismutase [Mn]	0.096	-0.193	0.073	-0.331	0.078	0.102	0.174	0.150	44.98	9.19	77.57	7.71	0.259	0.104	0.682	0.064	4	5	6	5	6
Q3UGZ4_MOUSE	SPNB3	unnamed protein product	-0.024	-0.062	-0.053	-0.125	0.059	0.059	0.118	0.052	68.71	43.44	70.13	37.08	0.697	0.319	0.695	0.048	17	40	37	33	24
SPTA2_MOUSE	SPTAN1	Spectrin alpha chain, brain	-0.030	-0.110	-0.032	-0.114	0.054	0.039	0.104	0.071	66.90	12.05	74.85	56.85	0.605	0.029	0.793	0.148	106	131	125	119	106

SPTB2_MOUSE	SPTBN1	Spectrin beta chain, brain 1	-0.033	-0.058	0.014	-0.048	0.038	0.044	0.086	0.054	66.90	42.52	84.42	66.46	0.411	0.220	0.890	0.407	60	90	88	92	75
SRCN1_MOUSE	SRCIN1	p130Cas-associated protein	-0.089	-0.070	0.081	0.042	0.103	0.052	0.085	0.096	62.80	41.79	77.57	52.60	0.413	0.213	0.383	0.673	2	19	10	15	12
STX1A_MOUSE	STX1A	Syntaxin-1A	-0.111	-0.242	0.027	-0.238	0.059	0.052	0.123	0.095	42.55	0.00	84.42	11.02	0.097	0.002	0.848	0.039	5	9	8	9	6
STX1B_MOUSE	STX1B	Syntaxin-1B	-0.054	-0.168	0.012	-0.211	0.065	0.037	0.072	0.076	66.90	2.48	84.42	10.42	0.432	0.004	0.890	0.024	12	15	14	15	14
STXB1_MOUSE	STXBP1	Syntaxin-binding protein 1	-0.030	-0.015	0.040	-0.021	0.038	0.044	0.041	0.043	66.90	>100.00	81.76	>100.00	0.461	0.738	0.346	0.636	20	30	22	23	20
SUCA_MOUSE	SUCLG1	Succinyl-CoA ligase [GDP-forming] subunit alpha	0.109	-0.051	0.017	-0.238	0.083	0.067	0.105	0.060	38.56	46.49	84.42	2.97	0.225	0.476	0.868	0.008	5	8	7	5	5
SV2A_MOUSE	SV2A	Synaptic vesicle glycoprotein 2A	-0.011	0.050	0.090	-0.154	0.055	0.061	0.093	0.028	69.52	33.34	77.57	10.15	0.852	0.439	0.367	0.001	6	13	11	13	9
SV2B_MOUSE	SV2B	Synaptic vesicle glycoprotein 2B	-0.061	0.006	0.025	-0.166	0.036	0.056	0.075	0.043	51.02	>100.00	84.42	11.02	0.136	0.913	0.773	0.005	9	9	9	8	9
SYN1_MOUSE	SYN1	Synapsin-1	-0.085	-0.365	0.000	-0.499	0.042	0.030	0.081	0.093	42.55	0.00	>100.00	0.00	0.102	2.084	0.996	0.001	15	18	19	18	17
SYN2_MOUSE	SYN2	Synapsin-2	-0.062	-0.396	-0.122	-0.308	0.038	0.074	0.103	0.062	51.02	0.00	54.74	0.00	0.146	0.001	0.278	0.001	10	13	13	11	11
SYNJ1_MOUSE	SYNJ1	Synaptojanin-1	-0.017	-0.248	0.009	-0.073	0.054	0.035	0.121	0.095	69.52	0.00	84.08	65.26	0.757	0.001	0.949	0.467	3	6	2	7	3
SYNPR_MOUSE	SYNPR	Synaptoporin	-0.067	0.104	0.126	0.097	0.141	0.083	0.116	0.083	66.90	21.11	77.57	42.52	0.653	0.248	0.357	0.277	2	5	3	3	2
SYPH_MOUSE	SYP	Synaptophysin	-0.023	-0.331	0.023	-0.339	0.056	0.071	0.118	0.088	68.53	0.00	84.42	0.00	0.691	0.002	0.865	0.012	4	6	6	6	7
SYT1_MOUSE	SYT1	Synaptotagmin-1	-0.128	0.018	0.098	-0.125	0.059	0.056	0.074	0.043	36.04	>100.00	77.57	30.51	0.062	0.754	0.258	0.020	9	11	12	11	9
THY1_MOUSE	THY1	Thy-1 membrane glycoprotein	-0.047	-0.117	-0.005	-0.528	0.105	0.085	0.089	0.203	67.56	34.22	>100.00	0.00	0.668	0.208	0.963	0.047	6	6	5	5	5
TKT_MOUSE	TKT	Transketolase	-0.134	0.084	0.053	0.207	0.083	0.056	0.102	0.074	50.18	21.11	81.64	21.15	0.144	0.190	0.598	0.027	4	8	8	6	8
TENR_MOUSE	TNR	Tenascin-R	-0.031	0.202	0.297	-0.022	0.061	0.078	0.183	0.138	67.56	6.27	77.57	>100.00	0.628	0.032	0.131	0.878	9	16	15	12	9
TOM70_MOUSE	TOMM70A	Mitochondrial import receptor subunit TOM70	0.191	0.014	-0.061	0.177	0.103	0.058	0.101	0.106	25.67	>100.00	70.39	33.94	0.101	0.811	0.563	0.137	5	10	7	9	8
TPIS_MOUSE	TPI1	Triosephosphate isomerase	-0.048	-0.096	-0.172	0.190	0.085	0.058	0.250	0.171	66.90	34.22	54.74	33.94	0.588	0.143	0.509	0.301	8	9	7	10	9
TPPP_MOUSE	TPPP	Tubulin polymerization-promoting protein	-0.062	-0.100	0.021	-0.290	0.073	0.072	0.106	0.069	66.90	36.56	84.42	0.00	0.423	0.205	0.850	0.005	6	9	6	9	6
TTYH1_MOUSE	TTYH1	Protein tweety homolog 1	0.039	0.180	0.227	0.267	0.083	0.109	0.172	0.150	74.06	13.62	77.57	27.41	0.656	0.139	0.263	0.113	2	3	3	3	3
TBB3_MOUSE	TUBB3	Tubulin beta-3 chain	-0.193	-0.546	-0.230	-0.505	0.122	0.125	0.357	0.207	48.32	0.00	48.33	0.00	0.171	0.003	0.549	0.041	3	3	3	5	2
UCHL1_MOUSE	UCHL1	Ubiquitin carboxyl-terminal hydrolase isozyme L1	-0.122	-0.169	-0.252	0.204	0.108	0.079	0.221	0.150	53.37	9.19	48.33	33.94	0.293	0.072	0.289	0.213	3	5	3	2	5
QCR7_MOUSE	UQCRB	Cytochrome b-c1 complex subunit 7	0.193	0.067	-0.191	-0.097	0.098	0.104	0.198	0.146	25.67	33.34	48.33	65.26	0.086	0.547	0.390	0.527	5	9	7	7	6
QCR1_MOUSE	UQCRC1	Cytochrome b-c1 complex subunit 1	0.166	0.063	-0.172	-0.077	0.090	0.102	0.190	0.115	25.67	33.34	48.33	65.26	0.104	0.557	0.400	0.526	6	10	10	9	10
QCR2_MOUSE	UQCRC2	Cytochrome b-c1 complex subunit 2	0.167	0.105	-0.192	-0.041	0.087	0.099	0.173	0.130	25.67	21.11	48.33	>100.00	0.090	0.327	0.340	0.767	9	14	13	15	14
UCRI_MOUSE	UQCRFS1	Cytochrome b-c1 complex subunit Rieske	0.156	0.104	-0.283	0.045	0.084	0.112	0.206	0.099	25.67	22.83	48.33	52.60	0.113	0.379	0.214	0.661	4	5	3	5	5
TERA_MOUSE	VCP	Transitional endoplasmic reticulum ATPase	0.018	-0.055	0.027	-0.008	0.119	0.038	0.120	0.084	76.98	42.52	84.42	>100.00	0.886	0.195	0.821	0.929	4	5	5	4	6
VDAC1_MOUSE	VDAC1	Voltage-dependent anion-selective channel protein 1	0.158	0.175	-0.084	0.163	0.093	0.073	0.153	0.126	30.45	8.39	71.00	33.94	0.135	0.054	0.639	0.235	7	11	10	9	7
VDAC2_MOUSE	VDAC2	Voltage-dependent anion-selective channel protein 2	0.183	-0.038	-0.094	0.092	0.067	0.113	0.132	0.109	19.68	>100.00	70.88	51.06	0.026	0.747	0.487	0.429	5	8	5	7	7
VDAC3_MOUSE	VDAC3	Voltage-dependent anion-selective channel protein 3	0.152	0.079	-0.118	-0.006	0.067	0.075	0.106	0.122	25.67	24.72	54.74	>100.00	0.060	0.338	0.282	0.964	4	7	5	5	6
VISL1_MOUSE	VSNL1	Visinin-like protein 1	0.059	-0.144	-0.174	-0.140	0.073	0.064	0.146	0.135	58.53	9.59	48.33	59.40	0.445	0.061	0.290	0.334	5	5	6	4	5
1433B_MOUSE	YWHAB	14-3-3 protein beta/alpha	-0.250	-0.166	0.000	0.027	0.143	0.053	0.179	0.148	41.68	5.07	>100.00	>100.00	0.131	0.019	0.998	0.862	1	4	4	3	2
1433E_MOUSE	YWHAE	14-3-3 protein epsilon	0.010	-0.083	-0.190	0.071	0.058	0.085	0.203	0.156	76.98	42.52	48.33	51.06	0.862	0.371	0.386	0.660	8	10	9	8	7
1433G_MOUSE	YWHAG	14-3-3 protein gamma	-0.254	-0.076	-0.147	-0.013	0.077	0.082	0.223	0.159	14.31	43.44	54.74	>100.00	0.013	0.382	0.520	0.935	4	5	6	8	5
1433F_MOUSE	YWHAH	14-3-3 protein eta	0.088	-0.154	-0.081	0.023	0.022	0.111	0.195	0.141	20.99	26.36	69.92	>100.00	0.006	0.229	0.674	0.876	2	3	4	3	3
1433T_MOUSE	YWHAQ	14-3-3 protein theta	-0.189	0.001	-0.145	0.280	0.069	0.100	0.170	0.122	27.55	>100.00	54.74	21.42	0.042	0.991	0.443	0.072	1	4	2	3	3
1433Z_MOUSE	YWHAZ	14-3-3 protein zeta/delta	-0.028	-0.091	-0.270	0.182	0.062	0.070	0.227	0.169	68.31	40.16	48.33	33.94	0.666	0.242	0.295	0.315	6	7	8	10	8
148686951		mCG145297	-0.142	0.078	0.172	-0.046	0.142	0.126	0.090	0.163	55.48	33.34	77.57	>100.00	0.360	0.560	0.085	0.788	2	4	4	5	2
148690766		mCG5710	0.009	-0.085	0.073	-0.069	0.066	0.047	0.102	0.082	76.98	35.97	77.57	65.26	0.893	0.106	0.485	0.432	2	5	4	5	6
