# Supplementary material for: Reducing hippocampal extracellular matrix reverses early memory deficits in a mouse model of Alzheimer’s disease
Source: Acta Neuropathol Commun. 2014 Jun 29;2:76. doi: 10.1186/s40478-014-0076-z (PMC4149201; doi:10.1186/s40478-014-0076-z)
Supplement: Additional file 4: Figure S2. — APP/Aβ levels at hippocampal synaptic sites are significantly increased in APP/PS1 mice. a Proteomics analysis revealed a significant increase in APP levels at hippocampal synaptic sites in APP/PS1 transgenic (TG) mice compared with wildtype (WT) controls at 3, 6 and 12 months of age; n = 5 mice per genotype (SAM analysis; mean ± SEM; *FDR < 10). b Increased levels of APP as detected by proteomics analysis are primarily due to an increase in the levels of the Aβ-specific peptide LVFFAEDVGSNK (indicated in red), in particular at 12 months of age. c The increase in synaptic Aβ levels is further confirmed by immunoblotting using the Aβ-specific antibody 6E10. An age-dependent accumulation of monomeric and low molecular weight oligomeric Aβ is observed, starting as early as 1.5 months of age. [file 40478_2014_76_MOESM4_ESM.pdf]

Reducing hippocampal extracellular matrix reverses early memory deficits in a mouse model of Alzheimer's disease

Marlene J. Végh[a], Céline M. Heldring[a], Willem Kamphuis[b], Sara Hijazi[a], Arie J. Timmerman[a], Ka Wan Li[a], Pim van Nierop[a], Huibert D. Mansvelder[a], Elly M. Hol[c], August B. Smit[a], Ronald E. van Kesteren[a]

[a]Center for Neurogenomics and Cognitive Research, Neuroscience Campus Amsterdam, VU University, De Boelelaan 1085, 1081 HV Amsterdam, The Netherlands.

[b]Netherlands Institue for Neuroscience, Meibergdreef 47, 1105 BA Amsterdam, The Netherlands.

[c]Department of Translational Neuroscience, Brain Center Rudolf Magnus, University Medical Center Utrecht, Universiteitsweg 100, 3584 CG, Utrecht, The Netherlands.

Correspondence should be addressed to R.E. van Kesteren. E-mail: ronald.van.kesteren@vu.nl, phone: +31205987111, fax: +31205989281.

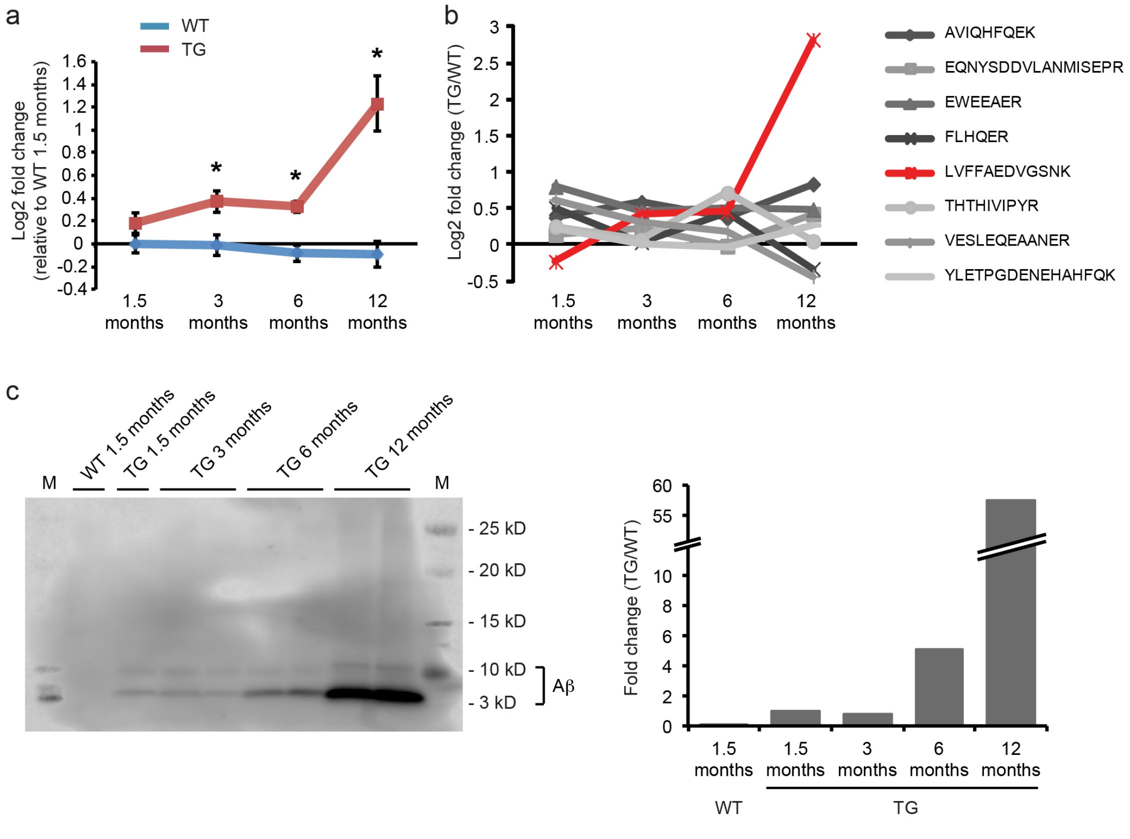

Suppl. Fig. 2 APP/Aβ levels at hippocampal synaptic sites are significantly increased in APP/PS1 mice. **a** Proteomics analysis revealed a significant increase in APP levels at hippocampal synaptic sites in APP/PS1 transgenic (TG) mice compared with wildtype (WT) controls at 3, 6 and 12 months of age; n = 5 mice per genotype (SAM analysis; mean ± SEM; *FDR < 10). **b** Increased levels of APP as detected by proteomics analysis are primarily due to an increase in the levels of the Aβ-specific peptide LVFFAEDVGSNK (indicated in red), in particular at 12 months of age. **c** The increase in synaptic Aβ levels is further confirmed by immunoblotting using the Aβ-specific antibody 6E10. An age-dependent accumulation of monomeric and low molecular weight oligomeric Aβ is observed, starting as early as 1.5 months of age
