# Supplementary material for: Reducing hippocampal extracellular matrix reverses early memory deficits in a mouse model of Alzheimer’s disease
Source: Acta Neuropathol Commun. 2014 Jun 29;2:76. doi: 10.1186/s40478-014-0076-z (PMC4149201; doi:10.1186/s40478-014-0076-z)
Supplement: Additional file 5: Table S2. — Functional enrichment of differentially expressed proteins at 3 months of age. Significantly enriched (Fisher’s exact p-value <0.05) GO terms are listed. Enriched GO terms were assigned to two functional groups: extracellular matrix and presynaptic neurotransmitter release. [file 40478_2014_76_MOESM5_ESM.pdf]

# Reducing hippocampal extracellular matrix reverses early memory deficits in a mouse model of Alzheimer's disease

**Marlene J. Végh[a], Céline M. Heldring[a], Willem Kamphuis[b], Sara Hijazi[a], Arie J. Timmerman[a], Ka Wan Li[a], Pim van Nierop[a], Huibert D. Mansvelder[a], Elly M. Hol[c], August B. Smit[a], Ronald E. van Kesteren[a]**

[a]Center for Neurogenomics and Cognitive Research, Neuroscience Campus Amsterdam, VU University, De Boelelaan 1085, 1081 HV Amsterdam, The Netherlands.

[b]Netherlands Institue for Neuroscience, Meibergdreef 47, 1105 BA Amsterdam, The Netherlands.

[c]Department of Translational Neuroscience, Brain Center Rudolf Magnus, University Medical Center Utrecht, Universiteitsweg 100, 3584 CG, Utrecht, The Netherlands.

Correspondence should be addressed to R.E. van Kesteren. E-mail: ronald.van.kesteren@vu.nl, phone: +31205987111, fax: +31205989281.

## Supplementary Table 2

**Suppl. Table 2** Functional enrichment of differentially expressed proteins at 3 months of age. Significantly enriched (Fisher's exact *p*-value <0.05) GO terms are listed. Enriched GO terms were assigned to two functional groups: extracellular matrix and presynaptic neurotransmitter release

| GO term | Proteins | Fold enrichment | Fisher's exact *p*-value |
|---|---|---|---|
| **Extracellular matrix (DAVID enrichment score: 1.30)** | | | |
| GO:0031012~extracellular matrix | HAPLN1, SLC1A3, TNR, BCAN, NCAN | 4.4 | 0.0098 |
| GO:0005578~proteinaceous extracellular matrix | HAPLN1, SLC1A3, TNR, BCAN, NCAN | 4.4 | 0.0098 |
| GO:0001871~pattern binding | NCAM1, HAPLN1, APP, BCAN, NCAN | 3.7 | 0.0233 |
| GO:0030247~polysaccharide binding | NCAM1, HAPLN1, APP, BCAN, NCAN | 3.7 | 0.0233 |
| GO:0005539~glycosaminoglycan binding | NCAM1, HAPLN1, APP, BCAN, NCAN | 3.7 | 0.0233 |
| GO:0005576~extracellular region | HAPLN1, SLC1A3, PPIA, ADAM23, TNR, CFL1, BCAN, NCAN | 2.2 | 0.0434 |
| **Presynaptic neurotransmitter release (DAVID enrichment score: 0.91)** | | | |
| GO:0003001~generation of a signal involved in cell-cell signaling | RAB3A, STX1A, SYN1, SYNJ1, SYN2, CACNB4, RPH3A, SNAP25 | 2.7 | 0.0120 |
| GO:0007269~neurotransmitter secretion | RAB3A, STX1A, SYN1, SYNJ1, SYN2, RPH3A, SNAP25 | 2.8 | 0.0190 |
| GO:0016192~vesicle-mediated transport | RAB3A, STX1A, CLTB, NAPG, ICAM5, SYNJ1, NAPB, RPH3A, CLTC, STX1B, AMPH, SYP, PACSIN1, APP, ARF3, RAC1 | 1.7 | 0.0210 |
